# Activatable nanoflytrap achieves synergistic “Capture-Block-Regulate” therapy of pancreatic ductal adenocarcinoma

**DOI:** 10.1186/s12951-026-04420-3

**Published:** 2026-04-21

**Authors:** Kai Guo, Shuai Ren, Yingying Cao, Huifeng Zhang, Jin Cui, Li Xian Yip, Ximing Wang, Ying Tian, Lina Song, Zhongqiu Wang

**Affiliations:** 1 Department of Radiology, Affiliated Hospital of Nanjing University of Chinese Medicine, Nanjing, 210029 China; 2 Department of Radiology, Shandong Key Laboratory of Multimodal Imaging Artificial Intelligence and Genetics, Shandong Provincial Hospital Affiliated to Shandong First Medical University, Jinan, 250021 China; 3 Department of Chemical and Biomolecular Engineering, National University of Singapore, Singapore, 117585 Singapore

**Keywords:** Nanoflytrap, Dihydroartemisinin, Pancreatic ductal adenocarcinoma, Apoptosis, Ferroptosis

## Abstract

Pancreatic ductal adenocarcinoma (PDAC) remains one of the most refractory malignancies because of its dense stromal barrier and hypoxic microenvironment. Inspired by the Venus flytrap, we engineered an intelligent nanoflytrap (NT) system that performs a coordinated three stage therapeutic strategy of capture, block, and regulate for precise and sequential treatment. In the capture stage, the CGKRK containing tumor homing peptide enables dual recognition of p32 receptors that are overexpressed on tumor vasculature and PDAC cells, ensuring selective accumulation within the tumor microenvironment. The ROS responsive PLGA-TK-PEI copolymer further guarantees confined activation under oxidative stress. During the block stage, ROS triggered disassembly of NT releases the pro apoptotic peptide _D_(KLAKLAK)_2_, which disrupts mitochondrial membranes, induces apoptosis, and damages tumor vasculature, resembling the rapid closure of a flytrap. In the regulate stage, dihydroartemisinin undergoes Fe^2+^ catalyzed cleavage to generate reactive oxygen species, triggering ferroptosis and mitigating hypoxia induced resistance. Gadolinium integration facilitates MRI guided monitoring. Transcriptomic and metabolomic analyses reveal that NT orchestrates apoptosis and ferroptosis while disturbing lipid metabolism and suppressing angiogenesis through ROS accumulation. This biomimetic capture block regulate strategy effectively overcomes stromal and hypoxic barriers, providing a promising MRI guided theranostic platform for PDAC.

**Graphical Abstract:**

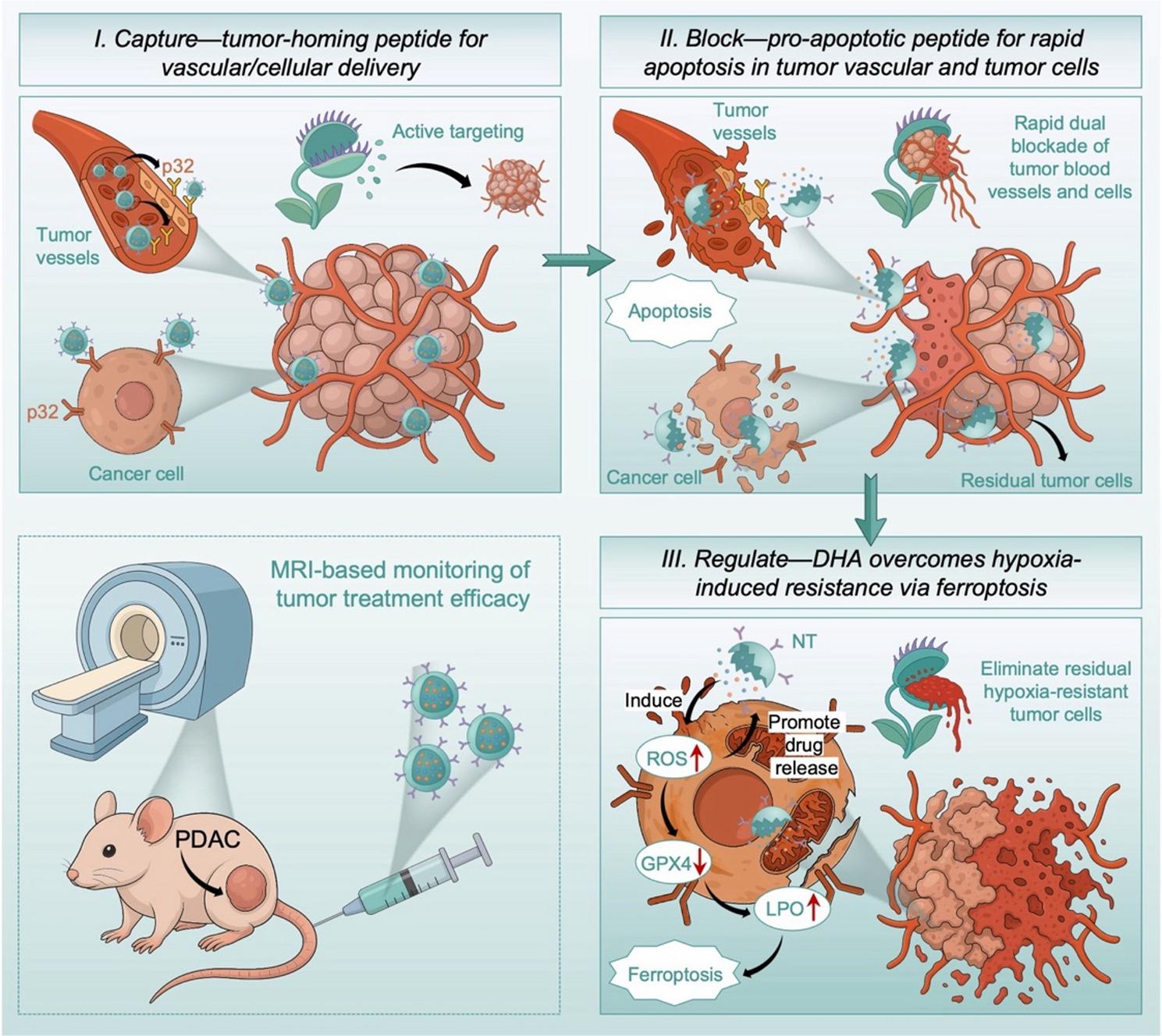

**Supplementary Information:**

The online version contains supplementary material available at 10.1186/s12951-026-04420-3.

## Introduction

Pancreatic ductal adenocarcinoma (PDAC) presents a significant clinical challenge due to its unique histoarchitectural characteristics, such as a dense extracellular matrix, disorganized vascular networks, and varying oxygen levels. These features collectively form a formidable biophysical barrier that hinders the effective delivery of conventional chemotherapy and targeted therapies [1–3]. Despite the initial promise of anti-angiogenic therapy in targeting tumor blood vessels, clinical outcomes in PDAC have been disappointing [4, 5].This therapeutic setback is attributed to several interconnected factors: disruption of blood vessels leading to severe hypoxia, which paradoxically promotes tumor aggressiveness through HIF-1α-mediated pathways, and the development of adaptive resistance by residual cancer cells through alternative angiogenic mechanisms [6, 7].Therefore, an integrated therapeutic strategy capable of simultaneously targeting tumor vasculature and cancer cells, while counteracting hypoxia-driven resistance, is urgently needed.

Inspired by the Venus flytrap’s stimulus-responsive prey capture mechanism, we developed an activatable nanoflytrap system that executes a synergistic “*Capture-Block-Regulate*” therapeutic sequence in response to the PDAC microenvironment. The plant’s mechanosensitive trigger hairs precisely detect insect contact through specialized stimulus-response mechanisms, enabling rapid prey capture only when specific mechanical thresholds are met [8]. Inspired by this biological precision, the reactive oxygen species (ROS)-responsive thioketal (TK)-modified copolymer (Poly (lactic-co-glycolic acid)-TK-Polyethylenimine, PLGA-TK-PEI) functions as an intelligent nanocarrier. Its ROS-labile cleavage property enables it to serve as a “molecular antenna” for specific recognition and response to oxidative stress signals in the tumor microenvironment (TME) [9–11]. In PDAC’s characteristic oxidative niche, selective TK bond cleavage triggers a precisely orchestrated structural transformation cascade involving nanoparticle swelling, surface charge reversal, and controlled therapeutic payload release [12, 13]. Furthermore, the targeting peptide containing the CGKRK motif demonstrates selective binding affinity for p32 receptors overexpressed on both tumor vasculature and PDAC cells [14–16]. By conjugating this peptide with the ROS-responsive copolymer PLGA-TK-PEI, the resulting nanosystem achieves intelligent discrimination of tumor microenvironmental signatures through simultaneous ROS responsiveness and p32 recognition. The resulting microenvironment-triggered activation mechanism ensures precise therapeutic module deployment while maintaining exceptional tumor specificity and minimal systemic exposure.

Upon activation, the nanoflytrap initiates a *Block* step modeled after the rapid closure of the Venus flytrap. Functioning by disrupting the tumor vasculature while simultaneously accumulating in mitochondria, the cationic pro-apoptotic peptide _D_(KLAKLAK)_2_ induces rapid nutrient deprivation [17–19]. However, vascular blockade exacerbates intra-tumoral hypoxia, which can activate HIF-1α and suppress apoptotic efficacy [20, 21]. To address this limitation and regulate the compromised tumor microenvironment, we incorporated dihydroartemisinin (DHA). In the presence of Fe^2+^, DHA undergoes cleavage to generate ROS, disturb redox balance, deplete glutathione, inactivate glutathione peroxidase 4 (GPX4), and trigger ferroptosis [22, 23]. As a hypoxia-insensitive cell death pathway, ferroptosis effectively complements apoptosis in oxygen-deprived regions [24, 25]. Thus, by integrating p32-directed capture, peptide-mediated vascular blockade, and DHA-driven metabolic regulation, our biomimetic nanoflytrap enables a coordinated three-pronged strategy: capture, block, and regulate, to overcome the physical and adaptive resistance barriers of PDAC.

**Fig. 1:**
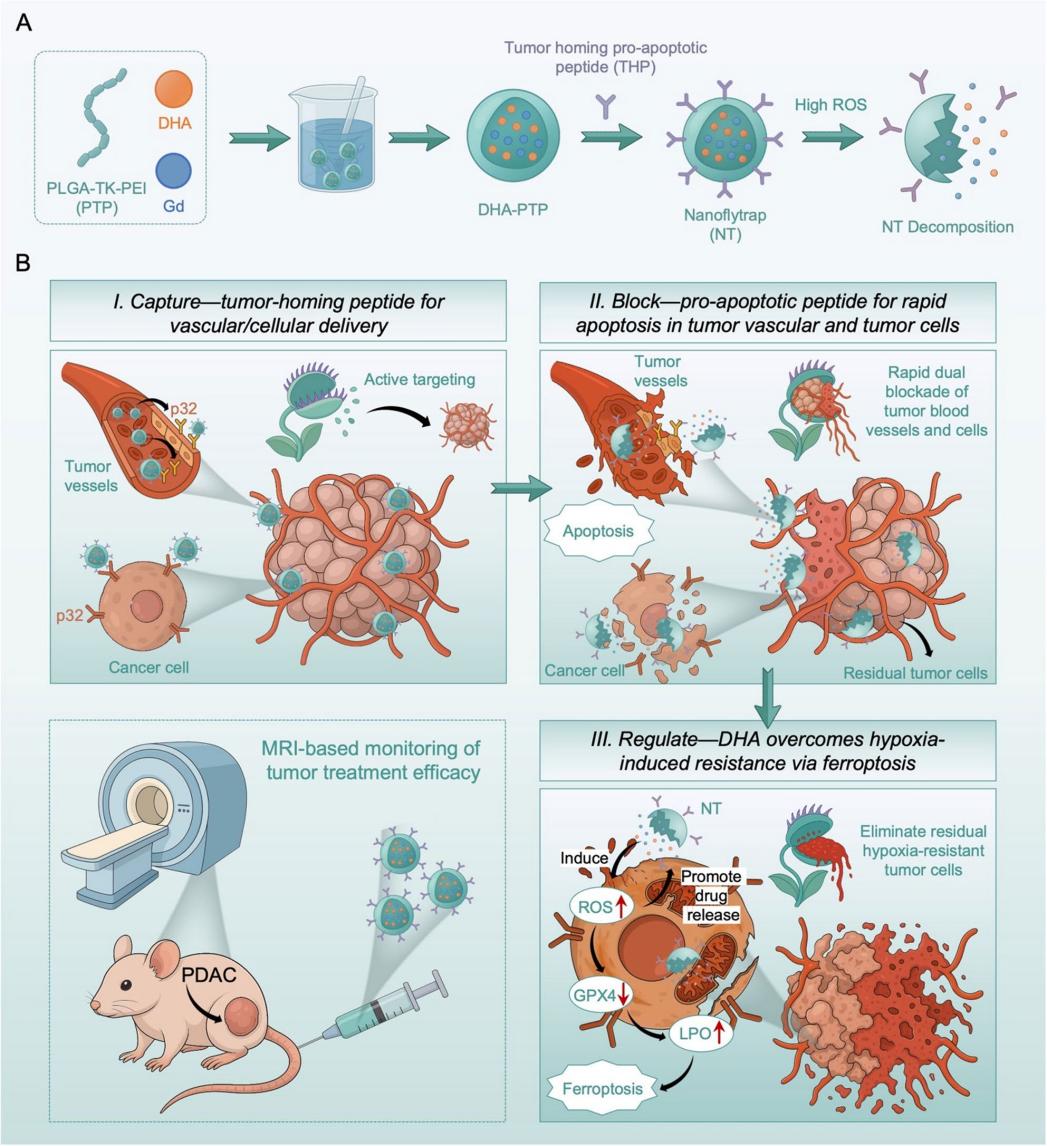
Illustration of **A** the ROS-responsive Venus-flytrap nanoflytrap (NT): PLGA-TK-PEI (PTP) is loaded with dihydroartemisinin (DHA) and gadopentetate dimeglumine (Gd) to form DHA-PTP, followed by conjugation with a tumor-homing pro-apoptotic peptide (THP). **B** Programmed tumor theranostics: (i) capture, (ii) block, and (iii) regulate under MRI guidance

Here, inspired by the stimulus-responsive prey-capture mechanism of the Venus flytrap, we propose a biomimetic “Capture–Block–Regulate” paradigm that transforms established functional modules into a programmed, microenvironment-driven therapeutic sequence. Rather than introducing new materials per se, this strategy emphasizes rational integration and temporal orchestration, enabling tumor-specific accumulation, vascular disruption–induced metabolic vulnerability, and subsequent hypoxia-insensitive ferroptosis induction. By aligning therapeutic actions with the evolving tumor microenvironment, this design aims to achieve synergistic efficacy that is unattainable through conventional co-delivery or additive treatment modalities (Fig. 1B).

## Results and discussion

### Synthesis and characterization of NT

**Fig. 2:**
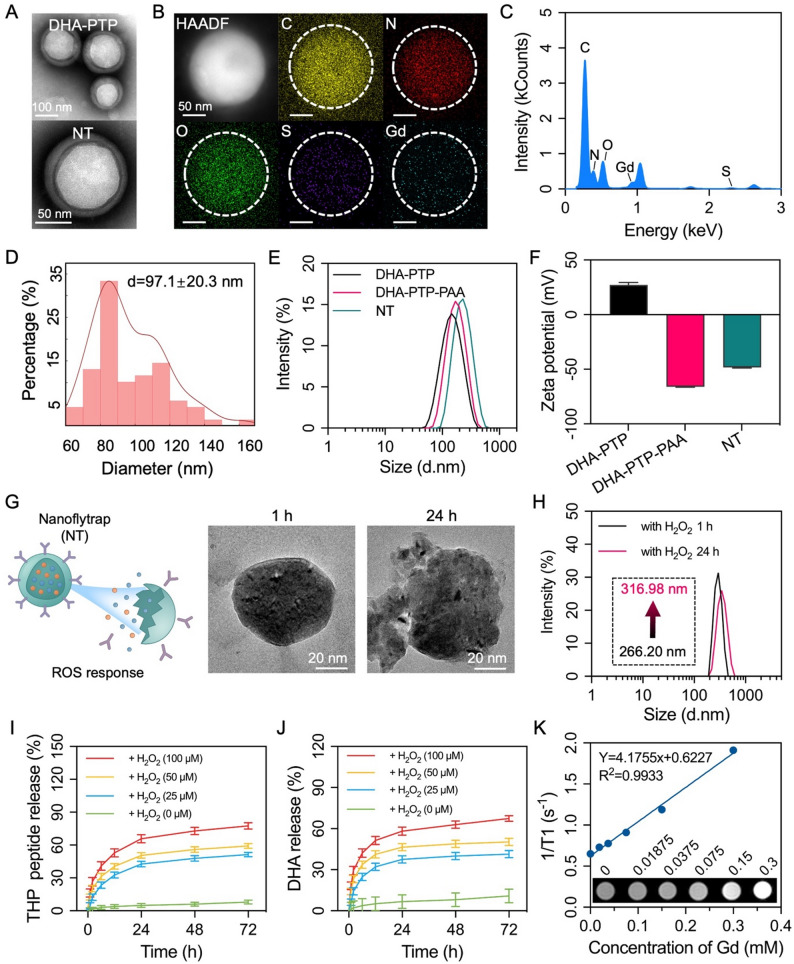
Characterization of NT. **A** Representative TEM images of DHA-PTP and NT. **B** Elemental mapping of NT. **C** EDS analysis. **D** Quantitative analysis of NT core diameter from TEM images. **E** Hydrodynamic diameter distributions of DHA-PTP, DHA-PTP-PAA, and NT by dynamic light scattering (DLS). **F** Zeta potential measurements of DHA-PTP, DHA-PTP-PAA, and NT. **G** TEM images and (**H**) DLS profiles of NT after H_2_O_2_ (100 µM) treatment for 1 h and 24 h. **I** THP peptide and (**J**) DHA cumulative release from NT. **K** MRI image and T1 relaxation rates of VNF hydrogels *in vitro*

Illustrated in Fig. 1A is the synthetic strategy employed for NT. Analysis using transmission electron microscopy (TEM) demonstrated that NT retained the morphological traits of the precursor DHA-PTP, presenting as spherical nanoparticles with an average diameter of 97.1 nm (Fig. 2A and D). Comprehensive Scanning Transmission Electron Microscopy-Energy Dispersive X-ray Spectroscopy (STEM-EDS) elemental mapping coupled with EDS spectroscopy unambiguously confirmed the presence of C, N, O, S, and Gd in NT (Fig. 2B and C). Size characterization by dynamic light scattering (DLS) demonstrated sequential increases in hydrodynamic diameters from 157.7 nm (DHA-PTP) to 184.4 nm (DHA-PTP- Poly (acrylic acid), DHA-PTP-PAA) and finally 239.7 nm (NT) (Fig. 2E), establishing the successful layer-by-layer assembly. The surface charge analysis revealed a remarkable transition from + 27.1 mV (DHA-PTP) to -65.9 mV (DHA-PTP-PAA) and − 48.3 mV (NT) (Fig. 2F), indicating effective surface modification. The hydrodynamic size and polydispersity index (PDI) of NT in serum exhibited no significant changes within 5 days, confirming the excellent stability of NT (Fig. S1). Surface negative charge improves pharmacokinetic profiles by enhancing colloidal stability, thereby extending the blood circulation half-life via reduced nonspecific clearance. Drug loading was verified by UV-Vis spectroscopy, where NT retained the characteristic absorption peaks of DHA (204 nm) and THP (240 and 497 nm) (Fig. S2). Quantitative analysis yielded encapsulation efficiencies of 62.6% for DHA and 38.1% for THP peptide. The ROS-responsive behavior was systematically investigated. Upon exposure to H_2_O_2_ (100 µM), TEM documented progressive nanoparticle swelling (Fig. 2G), while DLS tracked a gradual size increase (Fig. 2H), collectively demonstrating oxidative cleavage of thioketal linkers. Controlled release studies showed cumulative releases of 77.5% (THP) and 67.4% (DHA) at 72 h in H_2_O_2_ (100 µM) (Fig. 2I and J, Table S1). With increasing H_2_O_2_ concentration, the release of THP peptide and DHA both exhibited time dependence, confirming the responsiveness of NT to ROS. The release rate accelerated with elevated ROS concentration, indicating that the ROS-responsive behavior of NT is controllable and specific. Furthermore, NT exhibited concentration-dependent MRI signal enhancement (Fig. 2K) with an r_1_ relaxivity of 4.1755 mM^−1^s^− 1^, comparable to clinical Gd-based contrast agents. Collectively, these data confirm the successful fabrication of NT with optimal physicochemical properties for theranostic applications.

### NT for Tumor Targeting and Subcellular Localization

**Fig. 3:**
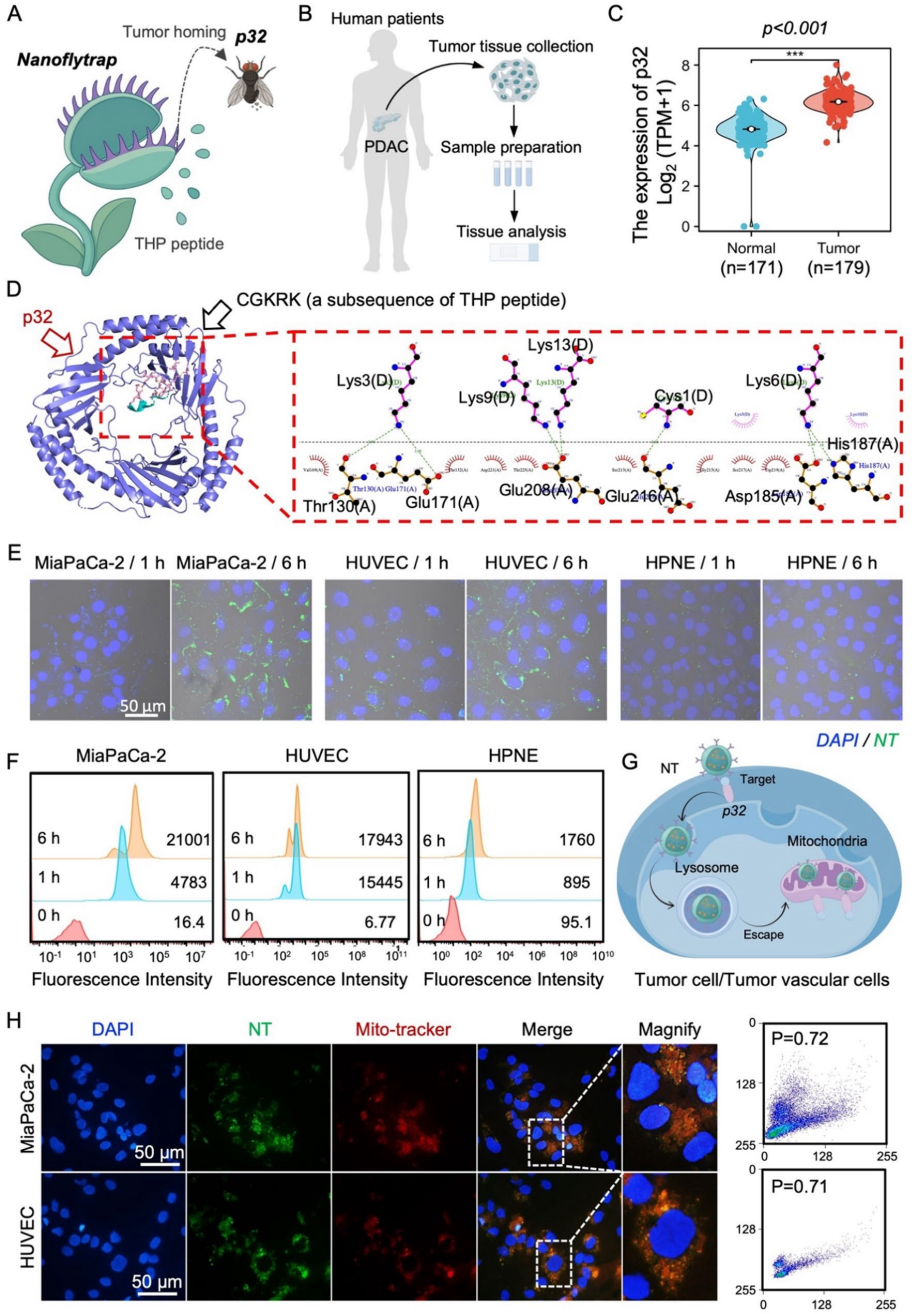
Venus-flytrap-inspired nanoflytrap (NT) for tumor targeting and subcellular localization. **A** Tumor-homing peptide-mediated targeting mimics the Venus flytrap predation mechanism. **B** Schematic workflow of clinical data integration with biospecimen collection/processing in PDAC cohort. **C** Comparative analysis of p32 expression between tumor and normal pancreatic tissues. ***p < 0.001. **D** Three-dimensional molecular docking analysis of CGKRK peptide fragment (a subsequence of the tumor-homing THP peptide) with p32 protein. **E** Confocal fluorescence imaging tracked NT internalization dynamics across different cell types (MiaPaCa-2, HUVEC, HPNE) following 1 h and 6 h of exposures. **F** Flow cytometric quantification of NT uptake in MiaPaCa-2, HUVEC, and HPNE cells. **G** Schematic of NT uptake. **H** Mitochondrial co-localization of NT in MiaPaCa-2 and HUVEC cells after 6 h of incubation, analyzed by *Pearson’s* correlation coefficient

Inspired by the insect-sensing targeting mechanism of the Venus flytrap, we developed a tumor-homing strategy leveraging the CGKRK (a subsequence of the tumor-homing THP peptide) (Fig. 3A). This peptide selectively binds to p32, a receptor highly expressed on tumor vasculature and cancer cell membranes. The validation of p32 as a dual-targetable biomarker in PDAC was performed through bioinformatics analysis of clinical datasets (Fig. 3B). As shown in Fig. 3C, p32 was significantly upregulated in PDAC tissues compared to normal pancreatic tissues. The potent, selective upregulation of p32 in tumor tissues strongly supports its potential as a theranostic target for precision medicine in PDAC.

Initial confirmation of p32 expression in pancreatic cancer cells and tumor vascular endothelial cells was performed using quantitative polymerase chain reaction (q-PCR). RNA samples were assessed for quality and concentration by gel electrophoresis and NanoDrop 2000 spectrophotometry. Clear and intact bands were observed in RNA electrophoresis, indicating satisfactory purity and concentration (Fig. S3), confirming the suitability of the extracted samples for subsequent PCR experiments. Q-PCR analysis revealed minimal p32 mRNA expression in normal pancreatic ductal epithelial cells (HPNE), while significantly higher expression was detected in pancreatic cancer cell lines (MiaPaCa-2, PANC-1, and BxPC-3) and tumor vascular endothelial cells (HUVEC) (Fig. S4). To further validate these findings, p32 protein expression was quantitatively examined by Western blot (WB) analysis (Fig. S5). Consistent with the q-PCR results, WB analysis validated elevated p32 protein levels in pancreatic cancer cells and tumor vascular endothelial cells, with MiaPaCa-2 cells showing the highest abundance. To assess the potential binding affinity between p32 and the targeting peptide, molecular docking simulations were performed. Protein-peptide docking simulations were conducted using AutoDock 4.2 software. Visualization analysis revealed that the active site of p32 was located at the position marked by a red arrow, while CGKRK was fully embedded within the structure, as indicated by a black arrow (Fig. 3D). A docking score below − 7 kcal/mol is generally indicative of strong binding activity between a small-molecule ligand and its protein target [26]. As shown in Table S2, the absolute docking scores for the top 10 conformations of p32-CGKRK complexes were exceptionally low, suggesting that CGKRK-modified NT possesses a high binding specificity for p32.

FITC was employed as a fluorescent tracer to monitor the cellular uptake efficiency of NT across different cell types. The cellular uptake kinetics of NT were evaluated in MiaPaCa-2, HUVECs, and HPNE cell lines using confocal laser microscopy at specific time points. A time-dependent increase in fluorescence intensity was observed with prolonged incubation. Notably, HPNE cells (p32-low-expressing) demonstrated significantly lower nanoparticle accumulation compared to p32-high-expressing cells (MiaPaCa-2 and HUVECs) at equivalent time points (Fig. 3E, S6), demonstrating the targeting specificity conferred by THP peptides modification. These qualitative findings were further corroborated by quantitative flow cytometry analysis (Fig. 3F).

The _D_(KLAKLAK)_2_ (a subsequence of the tumor-homing THP peptide), characterized by its strong positive charge, exerts cytotoxic effects through mitochondrial membrane depolarization. To elucidate the intracellular fate of NT, systematic colocalization analysis with key organelles was conducted using high-resolution fluorescence microscopy. The mitochondrial targeting efficiency was quantitatively assessed in MiaPaCa-2 and HUVEC cell lines. As shown in Fig. 3F, NT nanoparticles (green) exhibited pronounced colocalization with mitochondria (red), yielding *Pearson’s* correlation coefficients of 0.72 and 0.71 for MiaPaCa-2 and HUVEC, respectively [27]. These robust coefficients confirm the mitochondria-preferential accumulation of NT, likely facilitated by the peptide’s cationic properties. Parallel investigations of lysosomal involvement revealed even stronger colocalization patterns, with *Pearson’s* coefficients reaching 0.94 (MiaPaCa-2) and 0.91 (HUVEC) (Fig. S7). These findings demonstrate that following cellular internalization, NT undergoes lysosome-mediated trafficking and subsequent mitochondrial targeting driven by charge interactions, revealing a critical intracellular transport mechanism (Fig. 3G).

### Deep tumor penetration and targeted cytotoxicity of NT

**Fig. 4:**
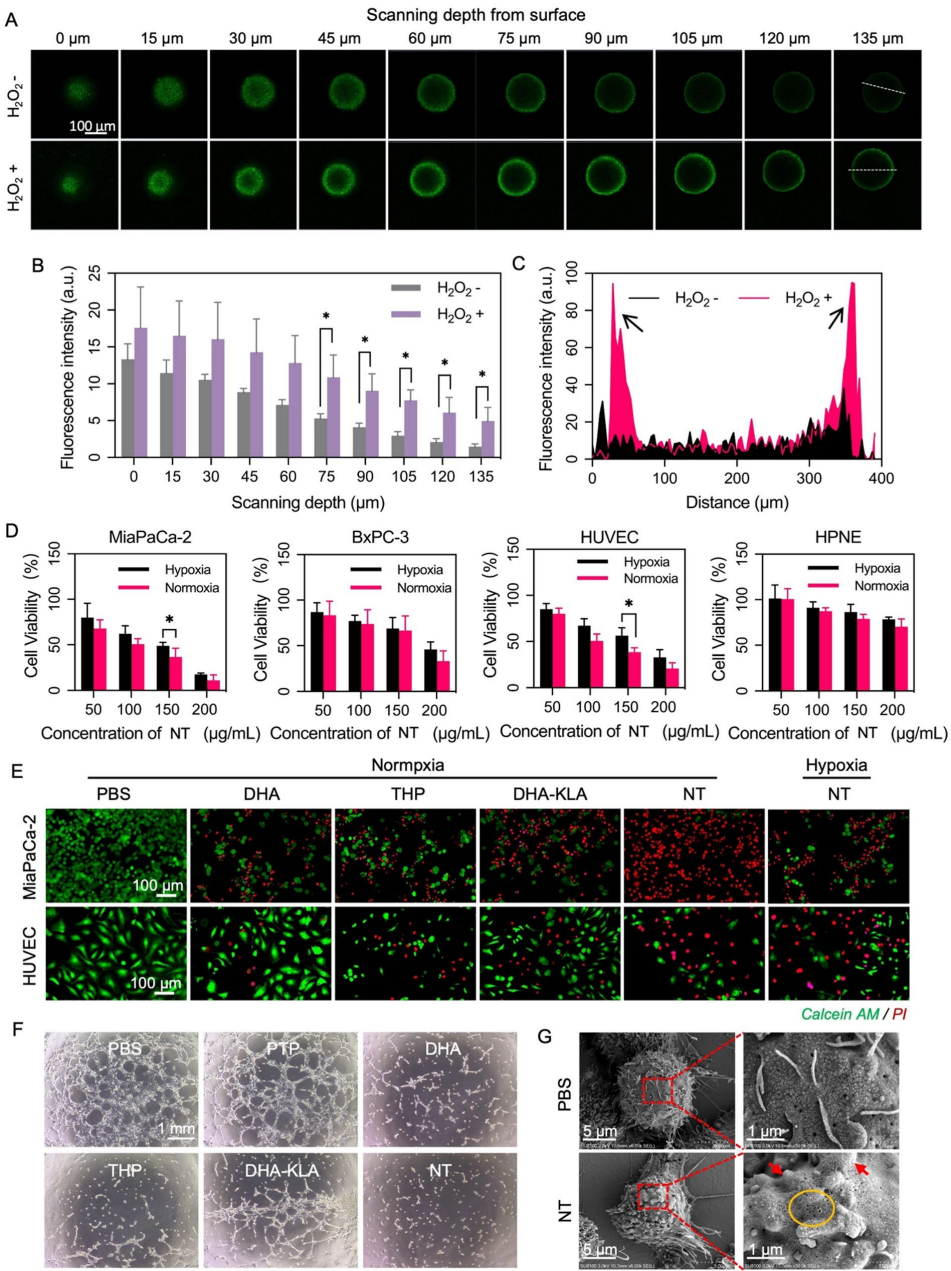
Deep tumor penetration and targeted cytotoxicity of NT. **A** Penetration of NT into MiaPaCa-2 multicellular tumor spheroids was assessed using Z-stack confocal laser scanning microscopy. **B** Quantitative analysis of the penetration depth of NT in three-dimensional tumor spheroid models. **C** Distribution of NT along the dotted white line at the depth of 135 μm in the cell sphere. **D** Concentration-dependent effects of NT on cellular proliferation under normoxia or hypoxia environments (*n* = 5). **E** Confocal microscopy images demonstrate live/dead staining of MiaPaCa-2 and HUVEC cells following various treatments. **F** Microscope images of tube formation of HUVEC cells after different treatments. **G** Scanning electron microscope images characterization of MiaPaCa-2 cells incubated with PBS or NT for 24 h. Red arrows indicate significant pseudopod retraction. Yellow circles demarcate membrane disintegration phenomena. *p < 0.05

Given the characteristically dense stromal architecture of PDAC that severely limits drug penetration, the ROS-responsive properties of NT were subsequently evaluated for their potential to enhance tumor penetration capability in dense 3D spheroid models mimicking PDAC desmoplasia. The spatial distribution of FITC-labeled NT fluorescence signals was examined using confocal laser scanning microscopy under different conditions. Notably, a marked enhancement in fluorescence intensity was observed in H_2_O_2_-treated 3D tumor spheroids (mimicking PDAC-TEM) compared to untreated controls (Fig. 4A). Upon ROS stimulation, NT undergoes structural swelling and charge reversal, facilitating deeper penetration into tumor spheroids. Quantitative analysis revealed fluorescence enhancement in NT + H_2_O_2_-treated samples versus controls, exhibiting fold-changes of 1.32 (15 μm), 1.44 (30 μm), 1.52 (45 μm), 1.61 (60 μm), 1.80 (75 μm), 2.05 (90 μm), 2.20 (105 μm), 2.63 (120 μm), and 2.92 (135 μm) (Fig. 4B). The spatial accumulation profile of NT at 135 μm penetration depth was further characterized in tumor spheroids (Fig. 4C). Under physiological conditions (H_2_O_2_-), no detectable fluorescence peaks were observed, whereas the simulated tumor microenvironment (H_2_O_2_+) elicited two distinct fluorescence maxima at localized positions of 23.4 μm and 358.7 μm, as indicated by black arrows, indicating the accumulation of NT. The observed spatiotemporal accumulation characteristics confirm the PLGA-TK-PEI carrier’s capacity to undergo redox-triggered structural transformation in tumor-mimetic conditions, thereby overcoming stromal diffusion barriers while maintaining efficient payload delivery to both peripheral and core tumor regions.

The cytotoxicity profiles of the individual components in NT (PLGA-TK-PEI, DHA, and THP peptide) and non-targeted nanoparticles (DHA-KLA) were systematically evaluated in MiaPaCa-2 and HUVEC cells (Fig. S8). The concentrations reported correspond to the total nanoparticle concentration, including both the PLGA-TK-PEI carrier and the encapsulated therapeutic agents. CCK-8 assays demonstrated excellent biocompatibility of the PLGA-TK-PEI carrier, maintaining > 90% cell viability even at 200 µg/mL, while DHA exhibited dose-dependent growth inhibition in both cell lines, confirming its therapeutic potential as a natural antitumor agent. THP peptide and DHA-KLA showed comparable antitumor efficacy. Furthermore, combination index analysis confirmed a synergistic interaction between DHA and THP (CI < 1, Fig. S9). NT treatment induced differential cytotoxicity across cell types under normoxia (Fig. 4D), with MiaPaCa-2 cells being the most sensitive (17.51 ± 3.74% viability at 200 µg/mL), followed by BxPC-3 (45.97 ± 8.05%) and HUVEC (33.02 ± 8.08%) cells, whereas normal HPNE cells had up to 78.37 ± 2.62% viability. This selective tumor targeting was further corroborated by live/dead staining (Fig. 4E, S10). Notably, hypoxia significantly attenuated NT cytotoxicity, likely through activation of drug-resistance pathways. Tumor angiogenesis critically supports cancer progression, making vascular targeting a promising therapeutic strategy. Tube formation assays revealed that neither PBS nor PLGA-TK-PEI impaired HUVEC network formation (Fig. 4F, S11), confirming the carrier’s biocompatibility. In contrast, DHA, THP, DHA-KLA, and NT treatments all disrupted tubular structure assembly, with NT exhibiting the most potent inhibition of total tube length. Scanning electron microscopy revealed distinct morphological alterations in NT-treated MiaPaCa-2 cells compared to PBS controls (Fig. 4G). Treated cells exhibited membrane blebbing (yellow circles) and pseudopod retraction (red arrows), indicating significant cellular damage. These findings confirm NT’s dual cytotoxic mechanism, exhibiting robust vascular disruption activity and direct tumor cell killing capacity.

### Mechanisms of NT-induced cytotoxicity

**Fig. 5:**
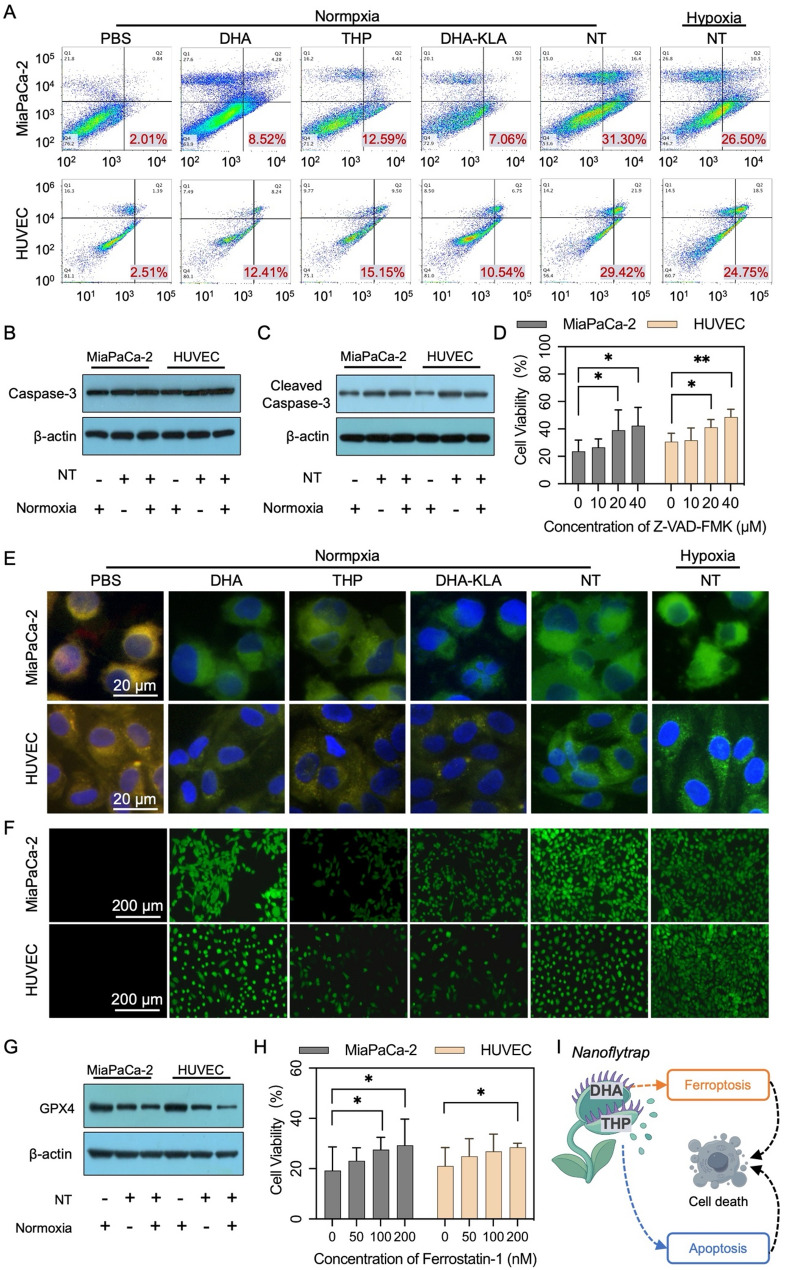
Mechanisms of NT-induced cytotoxicity. **A** Flow cytometric analysis of NT-mediated apoptosis in MiaPaCa-2 and HUVEC cells. **B**,** C** WB analysis of Caspase-3 and Cleaved Caspase-3 expression in MiaPaCa-2 and HUVEC cells under different treatment (*n* = 3). **D** Evaluation of NT-mediated toxicity in MiaPaCa-2 and HUVEC cells following pretreatment with the apoptosis inhibitor Z-VAD-FMK (*n* = 5). **E** JC-1 fluorescence imaging of mitochondrial membrane potential in MiaPaCa-2 and HUVEC cells after 24 h treatment under normoxic (21% O_2_) and hypoxic (1% O_2_) conditions. Red: J-aggregates (intact mitochondria), Green: Monomers (depolarized mitochondria). **F** Fluorescence imaging of ROS production in MiaPaCa-2 and HUVEC cells following drug treatments. Green: DCF fluorescence (ROS indicator). **G** Western blot analysis of GPX4 expression in MiaPaCa-2 and HUVEC cells with various treatments (*n* = 3). **H** Evaluation of NT-mediated toxicity in MiaPaCa-2 and HUVEC cells following pretreatment with the ferroptosis inhibitor ferrostatin-1 (*n* = 5). **I** Schematic illustration of dual cell death mechanisms induced by NT in tumor and vascular endothelial cells. *p < 0.05, **p < 0.01

The present findings demonstrate potent cytotoxic effects of NT against both pancreatic ductal adenocarcinoma cells (MiaPaCa-2) and tumor-associated vascular endothelial cells (HUVEC). To elucidate the underlying molecular mechanisms, a systematic evaluation of cell death pathways was performed. The _D_(KLAKLAK)_2_ peptide has been demonstrated to induce mitochondrial membrane permeabilization, followed by a loss of ΔΨm and culminating in the triggering of intrinsic apoptosis [18]. Flow cytometric analysis demonstrated that NT treatment significantly induced apoptosis, with total apoptotic rates reaching 31.30% in MiaPaCa-2 cells and 29.42% in HUVEC cells (Fig. 5A). Notably, although hypoxic conditions attenuated apoptotic responses, the pro-apoptotic activity maintained by NT was significantly higher than that induced by DHA or THP treatment alone, indicating a synergistic effect between the two components. Western Blot (WB) analysis confirmed NT-induced Caspase-3 activation, as evidenced by significantly increased levels of Cleaved Caspase-3 in both normoxic and hypoxic conditions compared to controls (Fig. 5B and C, S12). The apoptotic mechanism was further validated through dose-dependent rescue experiments, where pretreatment with the pan-caspase inhibitor Z-VAD-FMK restored cell viability (Fig. 5D). Additionally, time-dependent rescue analysis revealed that the protective effect of Z-VAD-FMK increased progressively over time and reached a plateau at approximately 24 h (Fig. S13). Beyond this time point, no further improvement in cell viability was observed, indicating that inhibition of apoptosis alone is insufficient to fully reverse NT-induced cytotoxicity at later stages.

The hypoxic nature of the PDAC’s TME and the consequent HIF-1α upregulation pose a fundamental limitation to the therapeutic efficacy of apoptosis-inducing strategies. DHA-mediated ferroptosis has emerged as a promising strategy to circumvent the treatment resistance imposed by hypoxia, addressing this critical clinical challenge. Mitochondrial membrane potential was assessed by JC-1 fluorescence staining. Compared to untreated controls, individually treatment with either DHA or THP reduced the red/green fluorescence intensity ratio in both MiaPaCa-2 and HUVEC cells (Fig. 5E, S14, S15), confirming mitochondrial dysfunction. Notably, NT treatment induced profound mitochondrial depolarization, with the red/green ratio significantly decreasing under both normoxic and hypoxic conditions, demonstrating oxygen-independent induction of mitochondrial damage. Mitochondria, which functioning as cellular “power plants”, play a pivotal role in energy metabolism and biosynthetic processes. Consequently, NT-induced mitochondrial depolarization triggers a bioenergetic crisis (Fig. S16) that disrupts macromolecular synthesis. WB analysis (Fig. S17) revealed that NT treatment significantly downregulated HIF-1α expression in PDAC cells under hypoxic conditions. This effect counteracted the characteristic hypoxia-induced upregulation of HIF-1α, demonstrating NT’s capacity to mitigate hypoxia-mediated therapeutic resistance. Intracellular ROS generation was quantified using the DCFH-DA fluorescence probe, revealing that NT-treated MiaPaCa-2 and HUVEC cells exhibited the most intense green fluorescence among all experimental groups (Fig. 5F, S18). The THP component alone also demonstrated ROS elevation, likely through disruption of mitochondrial electron transport chain integrity. This synergistic oxidative effect persisted under hypoxic conditions, confirming oxygen-independent ROS generation. The consequent lipid peroxidation (LPO) was assessed using the Liperfluo fluorescent probe, demonstrating that NT-treated cells exhibited the highest LPO levels among all experimental groups (Fig. S19). Intracellular glutathione (GSH) depletion was quantitatively assessed by measuring the GSH/GSSG ratio. A marked decrease in the GSH/GSSG ratio was triggered by NT treatment in MiaPaCa-2 cells, which was partially reversed by pretreatment with ferrostatin-1 (Fig. S20). WB analysis further demonstrated that NT downregulated GPX4 expression in MiaPaCa-2 and HUVEC cells compared to PBS controls (Fig. 5G, S21). The ferroptotic cell death mechanism was conclusively verified by dose-dependent attenuation of cytotoxicity with ferrostatin-1 (Fig. 5H). Time-dependent analysis further showed that the protective effect of Fer-1 gradually increased over time and reached a plateau at approximately 24 h (Fig. S22), with no additional recovery observed thereafter. This result indicates that ferroptosis cannot be completely reversed by inhibiting a single pathway. Collectively, these findings demonstrate that NT induces ferroptosis through coordinated GPX4 inhibition and GSH depletion-mediated lipid peroxidation. Although the above results demonstrate that NT is capable of inducing both apoptosis and ferroptosis, the critical question is whether these two pathways function independently or cooperatively in mediating NT-induced cytotoxicity. To address this issue, we performed dual-inhibition experiments to evaluate the functional interdependence between apoptosis and ferroptosis. In Fig.S23 Simultaneous inhibition of both apoptosis and ferroptosis led to a substantially greater rescue of cell viability in MiaPaCa-2 and HUVEC cells. The incomplete rescue observed with single-pathway inhibition, together with the pronounced recovery upon dual inhibition, demonstrates that apoptosis and ferroptosis function in a cooperative and interdependent manner rather than as parallel, independent pathways. This functional synergy is particularly relevant in the hypoxic PDAC microenvironment, where apoptosis alone is often attenuated and ferroptosis provides a complementary, oxygen-independent cell death route.

Bio-TEM analysis demonstrated characteristic morphological alterations in both MiaPaCa-2 and HUVEC cells following NT treatment, with simultaneous observation of apoptotic features (chromatin condensation, nuclear margination) and ferroptotic morphology (mitochondrial shrinkage, cristae disruption) (Fig. S24) [28, 29]. The co-existence of these distinct ultrastructural signatures provides direct visual evidence for NT’s unique capacity to coordinately induce both apoptosis and ferroptosis. These findings mechanistically demonstrate that the two active components of NT, DHA and THP, function synergistically, in a manner analogous to the twin lobes of a flytrap. DHA initiates lipid peroxidation to induce ferroptosis, while THP triggers mitochondria-mediated apoptosis; together, they ensure efficient elimination of both malignant cells and tumor-endothelial cells (Fig. 5I).

### In vivo biodistribution and tumor targeting efficiency of NT

**Fig. 6:**
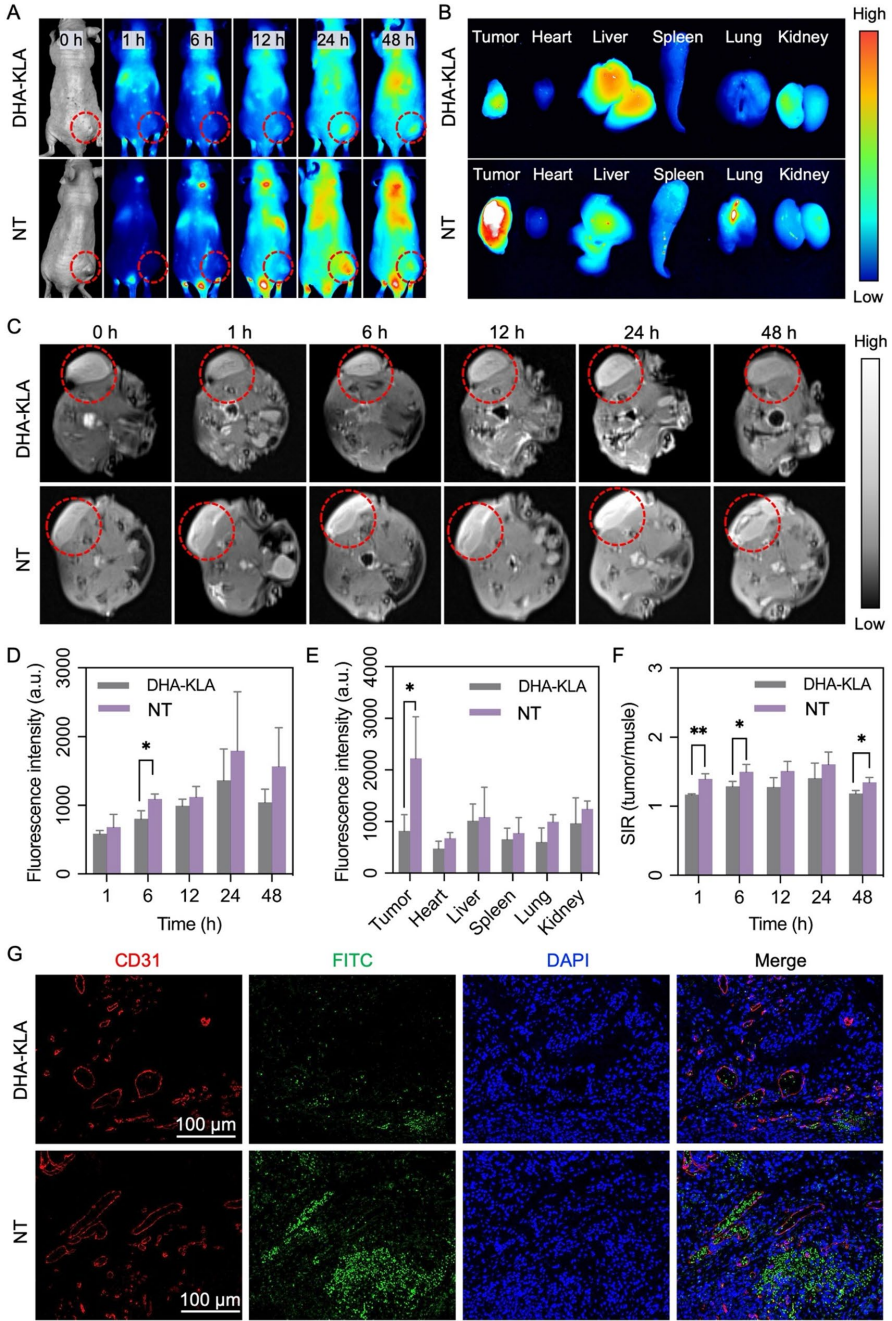
In vivo biodistribution and tumor targeting efficiency of NT. **A** Real-time near-infrared fluorescence imaging of tumor-bearing nude mice at indicated time points post intravenous injection of IR780-labeled NT (targeted) versus DHA-KLA (non-targeted) nanoparticles. **B** Ex vivo organ distribution 48 h post-injection, showing preferential tumor accumulation of NT. **C** T1-weighted MRI of tumor regions. **D** Quantitative fluorescence intensity analysis of tumor accumulation (*n* = 3). **E** Quantified fluorescence signals in major organs (*n* = 3). **F** Quantitative analysis of MRI contrast enhancement (*n* = 3). **G** Immunofluorescence co-localization analysis of tumor vasculature (CD31, red), nanoparticles (green), and nuclei (DAPI, blue) in tumor sections, demonstrating vascular extravasation and tumor penetration. *p < 0.05, **p < 0.01

To further investigate NT’s targeting specificity and tissue accumulation in vivo, near-infrared fluorescence imaging was employed to compare the biodistribution of targeted NT versus non-targeted DHA-KLA nanoparticles. For optimal in vivo optical detection, the nanocarriers were loaded with IR780, a long-wavelength fluorescent dye (ex/em: 780/830 nm) that provides enhanced tissue penetration depth and minimal autofluorescence interference (Fig. S25). As shown in Fig. 6A, real-time near-infrared fluorescence imaging revealed distinct temporal patterns of tumor accumulation (red circles) following intravenous administration of DHA-KLA or NT in tumor-bearing mice. Both nanoparticles exhibited peak fluorescence intensity at 24 h post-injection (Fig. 6D). However, NT demonstrated significantly prolonged circulation and enhanced tumor accumulation compared to DHA-KLA, confirming that CGKRK peptide modification improves tumor-targeting specificity and retention. Ex vivo organ imaging at 48 h further confirmed the differential distribution, with NT achieving a 2.5-fold greater tumor accumulation than DHA-KLA (Fig. 6B and E). Hepatic and renal fluorescence indicated liver and renal clearance pathways. It is noteworthy that fluorescence signals were also detected in the lungs. This is because a small amount of NT nanoparticles entering the bloodstream are engulfed by pulmonary macrophages, and the electrostatic interactions between the PLGA-TK-PEI core and the pulmonary endothelial cell membranes may further enhance NT retention in the lungs. The gadolinium-integrated NT nanoparticles functioned effectively as a contrast agent in MRI [30, 31], exhibiting time-dependent signal enhancement in tumor regions (red circles) that reached maximum intensity at 24 h post-administration, followed by gradual signal attenuation (Fig. 6C). Quantitative evaluation of tumor-to-muscle signal intensity ratios (SIR) demonstrated significantly greater values for NT compared to non-targeted DHA-KLA controls throughout the observation period (Fig. 6F), confirming the tumor-targeting advantages conferred by the CGKRK peptide modification.

To precisely characterize the spatial distribution patterns of NT within tumor tissues, high-resolution multiplex immunofluorescence imaging was performed. In Fig. 6G, it can be observed that FITC-labeled NT nanoparticles (green) demonstrated significantly enhanced tumor accumulation, greater distance from tumor vasculature (CD31, red), and broader distribution when contrasted with non-targeted DHA-KLA controls. These results provide definitive histological evidence that NT enhances tumor vascular extravasation, facilitates stromal barrier penetration, and promotes substantial drug accumulation in deep tumor tissues. Furthermore, we observed a reduction in CD31-positive microvessels in tumor tissues, which validates the vascular-block effect of NT.

### In vivo antitumor efficacy of NT

**Fig. 7:**
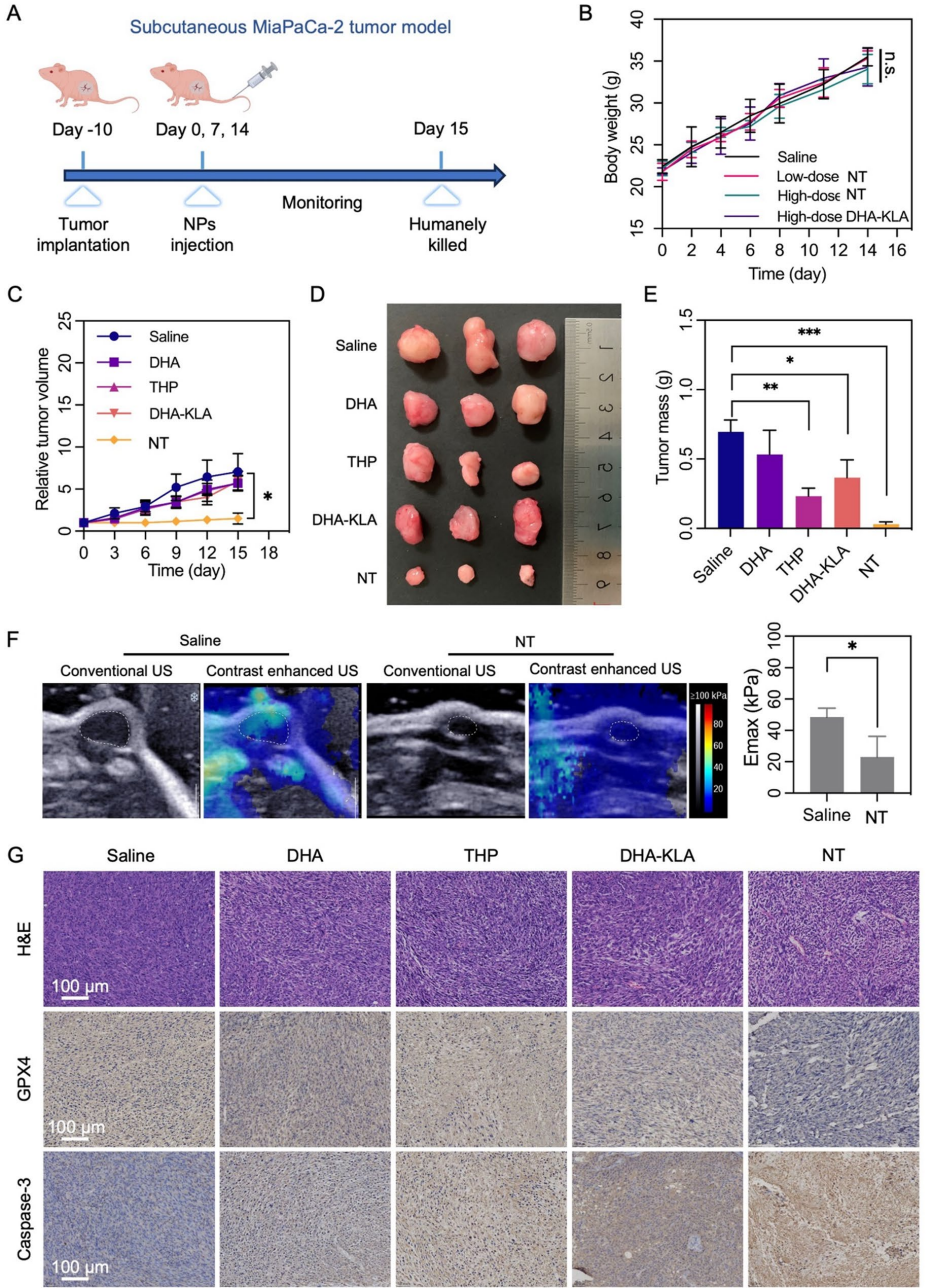
In vivo antitumor efficacy of NT. **A** Graphical abstract depicting the study design and treatment schedule. **B** Records of mouse body weight changes over the treatment course. **C** Tumor growth curves showing relative tumor volume over time (*n* = 3). **D** Tumor specimens collected from all experimental cohorts upon study termination are shown in these representative images (*n* = 3). **E** Quantitative analysis of tumor mass. **F** Ultrasound elastography and B-mode imaging demonstrating tumor stiffness changes across treatment groups (*n* = 3). Color bars indicate Young’s modulus values (kPa). **G** Histopathological and immunohistochemical analysis of tumor tissues. *p < 0.05, **p < 0.01, ***p < 0.001

NT demonstrates outstanding tumor-targeting and imaging capabilities, highlighting its significant potential for cancer theranostics. Figure 7A illustrates the specific therapeutic strategy. Body weight measurements revealed no statistically significant differences among the groups (Fig. 7B), confirming the excellent biocompatibility of NT. Notably, the NT group exhibited the smallest tumor mass and the lowest relative tumor volume compared to all control groups (Fig. 7C-E), demonstrating superior in vivo antitumor efficacy. Consistently, Kaplan–Meier analysis based on the time to a predefined humane endpoint (tumor volume ≥ 1000 mm^3^) showed that NT treatment significantly prolonged endpoint-free survival relative to the control groups (Fig. S26).

To further evaluate therapeutic outcomes, we employed ultrasound shear-wave elastography to assess changes in tumor tissue stiffness. On day 15 post-treatment, a significant difference in the maximum Young’s modulus (Emax) was observed between the saline control (48.6 ± 5.6 kPa) and NT-treated groups (23.1 ± 13.1 kPa) within the regions of interest (white dashed outlines, Fig. 7F). These results suggest that NT treatment alters the internal architecture and biomechanical properties of pancreatic cancer xenografts, reducing tissue stiffness-a finding consistent with tumor necrosis [32]. TdT-mediated dUTP Nick-End Labeling (TUNEL) staining confirmed this observation, revealing markedly elevated levels of necrotic cell death in tumor sections from the NT-treated group compared with the saline control (Fig. S27), further validating the necrotic effects of NT therapy *in vivo.*

Histopathological analysis *via* H&E staining revealed pronounced structural disruption and increased tumor cell porosity in the NT-treated group compared to controls (Fig. 7G). These findings align with the ultrasound shear-wave elastography results, which demonstrated a significant reduction in tumor stiffness, collectively indicating that NT induces extensive tumor necrosis. To further evaluate tumor microenvironment modulation, CD31 immunofluorescence staining demonstrated a significantly reduced vascular length density in NT-treated tumors compared with controls (Fig. S28), confirming vascular impairment in vivo. Interestingly, HIF-1α immunofluorescence analysis revealed a marked decrease in HIF-1α expression in the NT group (Fig. S29). Importantly, this reduction does not necessarily indicate improved oxygenation, but rather reflects suppression of hypoxia-adaptive signaling due to NT-induced mitochondrial dysfunction and extensive tumor cell death. To elucidate the underlying cell death mechanisms, we performed immunohistochemical analysis using ferroptosis-specific (GPX4) and apoptosis-specific (Caspase-3) markers. Figure 7G revealed a significant downregulation of GPX4 expression in DHA-, DHA-KLA-, and NT-treated tumors, with the most pronounced reduction observed in the NT group. In contrast, THP treatment did not induce statistically significant changes in GPX4 levels. Consistently, quantitative analysis using an malondialdehyde (MDA) and 4-hydroxynonenal (4-HNE) assay revealed a significant increase in lipid peroxidation following NT treatment, further confirming ferroptosis-associated lipid metabolic disruption (Fig. S30). These findings demonstrate that NT exhibits superior efficacy in triggering ferroptosis compared to other treatment modalities. Notably, Caspase-3 immunohistochemistry showed markedly enhanced staining intensity and significantly higher numbers of positive cells in NT-treated tumors relative to all other groups, confirming robust activation of apoptotic pathways. Collectively, these results establish that NT mediates potent antitumor effects through dual induction of both ferroptosis and apoptosis *in vivo.*

### Functional Mechanism of NT revealed by transcriptome and metabolome study

**Fig. 8:**
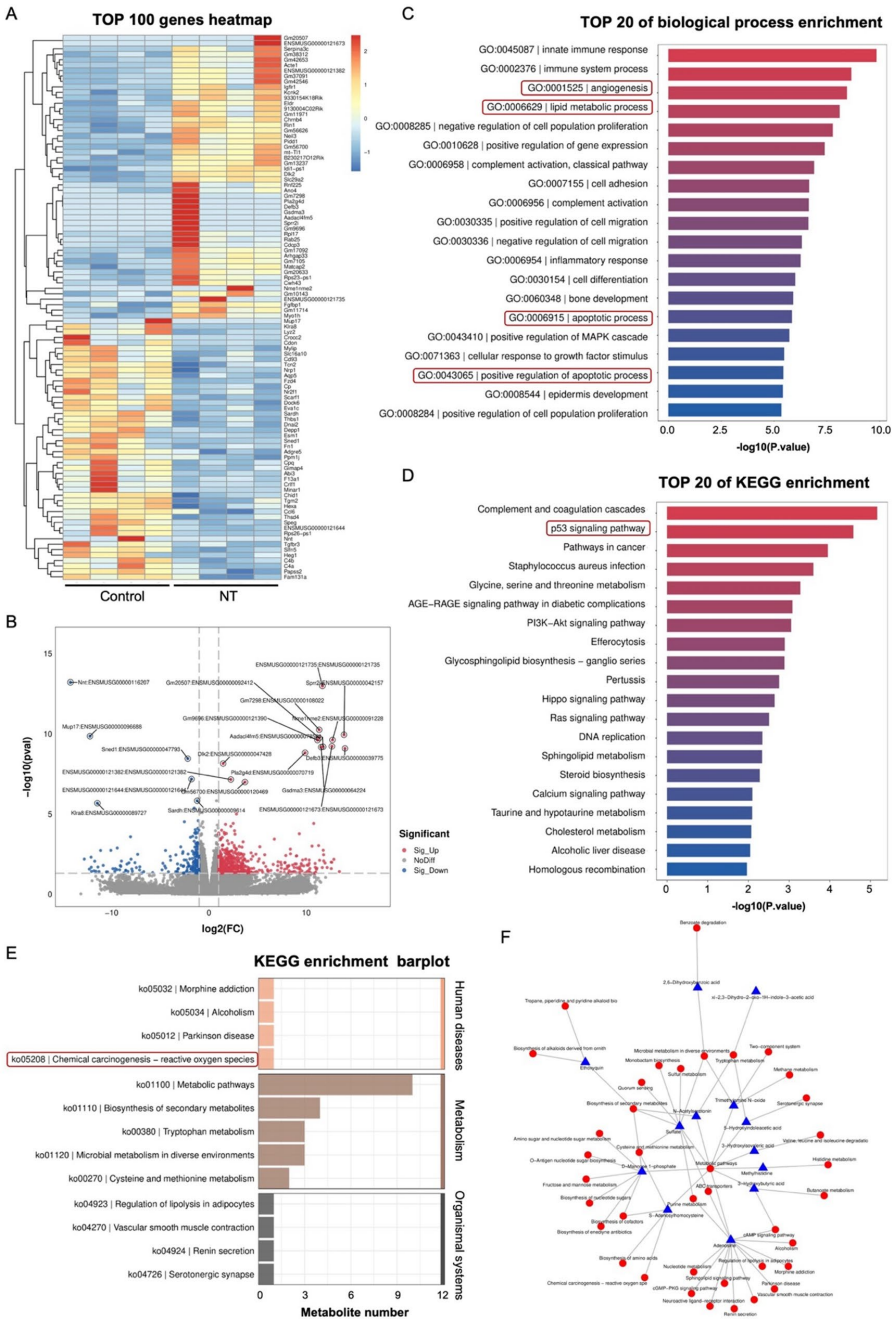
Transcriptomic and metabolomic analyses of tumors following NT treatment. **A** Heatmap of the top 100 DEGs between the NT-treated and control groups (*n* = 4). **B** Volcano plot of DEGs between the two groups; blue and red dots represent significantly downregulated and upregulated genes, respectively. **C** Top 20 biological processes identified by GO analysis. **D** Top 20 KEGG pathways. **E** KEGG category histogram of all significantly altered metabolites. **F** KEGG pathway-metabolite interaction network diagram

To gain deeper insights into the therapeutic and metabolic influence of NT, we performed tumor transcriptome sequencing against a reference genome alongside untargeted metabolomics. Relative to controls, a clustered heatmap of the 100 most significant functionally related DEGs reveals distinct expression patterns across experimental groups (Fig. 8A, Table S7). Furthermore, the distribution pattern of differentially expressed genes is displayed in volcano plots, which identified 798 upregulated and 836 downregulated genes under the combination treatment (Fig. 8B, S31, |log_2_FC| ≥ 1, *p* < 0.05).

Subsequently, GO enrichment analysis, which encompasses three major categories of Biological Process, Cellular Component, and Molecular Function, was performed to elucidate the functional changes, involvement, and activities of all significant differentially expressed genes. Analysis identified the most significantly enriched terms: 25 in Biological Process, 15 in Cellular Component, and 10 in Molecular Function (Fig. S32). To gain deeper insights into molecular functions, the 20 most statistically significant Biological Process terms were analyzed. The significant enrichment of apoptosis and positive regulation of apoptotic process strongly indicates that NT treatment directly activates programmed cell death, aligning with the principles of apoptotic therapy (Fig. 8C). This is further reinforced by the pronounced enrichment of the p53 signaling pathway (Fig. 8D), a master regulator of cell cycle arrest and apoptosis. This suggests that NT may exert its pro-apoptotic effects through activating p53-mediated transcriptional programs [33]. Notably, the alteration in lipid metabolic process offers a crucial mechanistic clue beyond apoptosis. Dysregulated lipid metabolism represents a recognized hallmark of ferroptosis, a non-apoptotic cell death pathway initiated by lipid peroxidation. Intriguingly, p53 also contributes to ferroptosis by regulating lipid metabolism and thereby downregulating SLC7A11, a critical element of the GPX4 antioxidant defense [34]. Therefore, the co-enrichment of lipid metabolic disruption and the p53 pathway implies that NT may induce ferroptosis in a p53-mediated manner, representing a synergistic cell death mechanism that could overcome apoptosis resistance [35, 36]. To validate the transcriptomic findings at the protein level, we performed WB analysis of key p53 downstream targets in tumor tissues. As shown in Fig. S33, NT treatment significantly increased the expression of phosphorylated p53, accompanied by upregulation of the pro-apoptotic proteins BAX and PUMA, and marked downregulation of SLC7A11. These results confirm activation of the p53 signaling pathway in vivo and support its involvement in NT-induced apoptosis and ferroptosis. Simultaneously, the suppression of angiogenesis suggests that NT inhibits the formation of new tumor vasculature, a mechanism central to vascular disruption therapy. The anti-angiogenic effect is also a well-established outcome of p53 activation, mediated through the transcriptional downregulation of pro-angiogenic factors like VEGF. Thus, the inhibition of angiogenesis observed here may be intrinsically linked to the activation of the p53 signaling pathway by NT, culminating in impaired nutrient supply and tumor regression [37].

Untargeted metabolomic profiling was performed to comprehensively characterize the metabolic alterations induced by NT treatment. A total of 371 upregulated and 612 downregulated differential metabolite ions were identified between the control and NT-treated groups (Fig. S34, |FC| ≥ 1.2, *p* < 0.05). The top 30 metabolites with the most significant changes are demonstrated in the clustering heatmap (Fig. S35). To decipher the functional implications of these metabolic alterations, KEGG pathway enrichment analysis was performed. The results identified several metabolic pathways significantly perturbed by NT intervention (Fig. 8E). Notably, the “Chemical carcinogenesis - reactive oxygen species” pathway was highly enriched. ROS serve as a central signaling node that can directly induce apoptosis by triggering mitochondrial membrane permeabilization and activating intrinsic apoptotic cascades [38]. Furthermore, ROS are fundamental executors of ferroptosis; excessive ROS production leads to iron-catalyzed lipid peroxidation-a hallmark of ferroptosis [39]. Pathway analysis results indicate that NT exposure leads to significant ROS induction, thereby simultaneously promoting both apoptotic and ferroptotic pathways. From the perspective of vascular disruption therapy, elevated ROS levels can severely damage endothelial cells, impair endothelial barrier function, and inhibit tumor angiogenesis [40]. Thus, the enrichment of the “Chemical carcinogenesis - reactive oxygen species” pathway implies that NT-induced ROS generation acts as a key upstream event, orchestrating a concerted antitumor response through activating multiple cell death programs and disrupting tumor vascular function. Finally, to integrate these findings and visualize the interactions between specific metabolites and pathways, a metabolite-pathway interaction network was constructed (Fig. 8F). This analysis revealed that key metabolites, such as methylhistidine, 3-hydroxyisovaleric acid, trimethylamine N-oxide, and acetylenedicarboxylic acid, function as hubs that concurrently modulate multiple enriched pathways. This network-based visualization intuitively illustrates the regulatory relationships between metabolites and pathways, suggesting that NT exerts its effects by coordinately disrupting a network of interconnected metabolic processes rather than targeting a single pathway. These findings indicate that NT acts as a multi-mechanistic therapeutic agent that simultaneously targets apoptosis, ferroptosis, and vascular support pathways, thereby orchestrating a coordinated antitumor response.

### Biocompatibility Assessment of NT

 In vitro hemolysis assays demonstrated that neither NT nor DHA-KLA induced significant hemolysis at various concentrations (Fig. S36), with all nanoprobe groups exhibiting hemolysis rates below 5% (Tables S3, S4), confirming their excellent hemocompatibility and compliance with biomedical nanomaterial safety standards [41]. Comprehensive biosafety evaluation revealed no statistically significant alterations in routine blood parameters or biochemical markers at 7- and 14- days post-administration compared to saline controls (Fig. S37, Tables S5 and S6), while histopathological examination of major organs showed no evidence of pathological abnormalities at either time point (Fig. S38). Collectively, these findings demonstrate that NT and DHA-KLA exhibit favorable biosafety profiles without inducing detectable toxicity in nude mouse models.

## Discussion

Effective targeted nanomedicine design requires not only prolonged systemic circulation and controlled drug release but also precise ligand–receptor recognition to ensure selective tumor accumulation while minimizing off-target exposure [42, 43]. The efficacy of targeted nanomedicines heavily relies on the specificity of the ligand-receptor interaction. Our utilization of the CGKRK peptide for p32 targeting offers distinct advantages over more commonly explored targeting moieties. While RGD-based systems achieve broad tumor targeting *via* αvβ3 integrin, their specificity for PDAC is limited due to integrin expression in stromal and normal tissues [44]. In contrast, p32 demonstrates pronounced overexpression specifically on PDAC cells and tumor vasculature, as validated by our bioinformatic and experimental data (Fig. 3B-D). This selectivity is reflected in the superior in vivo tumor accumulation of NT compared to the non-targeted DHA-KLA (Fig. 6A-E), yielding a 2.5-fold increase in fluorescence intensity. This performance surpasses that of many reported actively targeted nanosystems, such as folate-decorated liposomes, underscoring the clinical relevance of p32 as a target in PDAC [45]. In addition, iRGD (CRGDKGPDC) is a widely studied cyclic peptide and an emerging tumor-targeting ligand. It initially binds to integrin αvβ3/αvβ5 on tumor vasculature and subsequently undergoes proteolytic cleavage, exposing the C-end RGD motif and the C-terminal lysine–glycine–aspartic acid (KGD) sequence, thereby enabling tumor tissue penetration and secondary targeting of neuropilin-1 (NRP-1) on cancer cells [46]. However, the targeting of PDAC cells by iRGD is a secondary and indirect process: it first binds to integrins on tumor vessels, and only after proteolytic activation in the tumor microenvironment can the exposed KGD sequence interact with NRP-1 on PDAC cells. This two-step mechanism presents two limitations in the context of PDAC therapy. First, the efficiency of proteolytic activation is highly dependent on protease activity within the PDAC stroma, which is known to be heterogeneous and variable in clinical PDAC tissues. Second, the temporal delay inherent to secondary targeting is not optimal for our “Capture–Block–Regulate” strategy, which requires rapid vascular disruption and simultaneous targeting of cancer cells to ensure coordinated therapeutic execution. Therefore, p32 represents a more suitable and strategically consistent targeting ligand for our therapeutic approach.

In this study, we selected low–molecular weight PEI (10 kDa) and constructed a core–shell NT architecture (PLGA-TK-PEI core sequentially coated with PAA and CGKRK peptide) to mitigate potential immunogenicity. Compared with high–molecular weight PEI (≥ 25 kDa), which possesses a higher cationic charge density and readily interacts with plasma proteins such as complement components and immunoglobulins, 10 kDa PEI exhibits markedly reduced cytotoxicity and immunostimulatory potential [47]. Our in vitro biocompatibility studies confirmed minimal cytotoxicity (> 90% cell viability at 200 µg/mL) and negligible hemolysis (< 5%) (Fig. S33), supporting the safety profile of the selected PEI component. Furthermore, the anionic outer coating resulted in a final surface zeta potential of − 48.3 mV (Fig. 2F), effectively shielding the cationic core from direct interaction with circulating immune cells and plasma proteins, a well-established strategy to reduce the immunogenicity of cationic nanocarriers. Consistently, in vivo biodistribution studies showed no obvious accumulation in immune-related organs such as the spleen or liver (Fig. 6B and E), indirectly indicating the absence of significant nonspecific immune recognition or rapid clearance.

A critical challenge in PDAC is the dense desmoplastic stroma that severely limits drug diffusion. Our strategy employs an ROS-responsive PLGA-TK-PEI copolymer that undergoes structural transformation in the high-ROS tumor microenvironment, facilitating deep penetration. As mentioned, it is well-documented that ROS levels are significantly and selectively elevated in PDAC tissues compared with adjacent normal tissues [48]. This is primarily due to the heightened metabolic activity, sustained oxidative stress, and dysregulated redox homeostasis within the PDAC microenvironment. Tumor cells, including those in PDAC, generate higher levels of ROS as a result of abnormal metabolic pathways, including aerobic glycolysis (Warburg effect), which further contributes to the oxidative stress within the tumor niche. The selective difference in ROS levels between PDAC and normal tissues provides the biological basis for the ROS-responsive behavior of targeted probes and therapeutic agents. As shown in Fig. 4A-C, NT exhibited significantly enhanced distribution in 3D spheroids under H_2_O_2_ conditions, with fluorescence intensity increasing up to ~ 3-fold at a depth of 135 μm. This performance is notable when compared to other stimulus-responsive penetration strategies. For instance, MMP-cleavable peptides enhance penetration but are limited by variable protease expression [49]. pH-sensitive systems often trigger prematurely in the mildly acidic extracellular environment, leading to off-target release [50]. The ROS responsiveness of NT capitalizes on a hallmark feature of PDAC, elevated oxidative stress, ensuring spatially confined activation and release primarily within the tumor core. This mechanism, coupled with the carrier’s charge-reversal capability (facilitated by PEI protonation in lysosomes), promotes endolysosomal escape and intracellular delivery. Specifically, PEI contains abundant amine groups, which can undergo protonation in acidic environments, leading to a significant positive charge. This property is particularly important in the acidic environment of endosomes/lysosomes. Through this protonation, PEI enhances its interaction with the endosomal/lysosomal membrane, facilitating membrane disruption and promoting the release of the drug carrier into the cytoplasm. The positive charge of PEI not only aids in its interaction with the acidic endosomal membrane but also enhances the electrostatic attraction, leading to lysosomal membrane rupture and improving the endosomal escape of drug-loaded particles. Through this mechanism, PEI effectively prevents drug degradation in lysosomes, thereby improving therapeutic efficacy [51, 52].

The core therapeutic innovation of NT lies in the coordinated induction of apoptosis and ferroptosis. While numerous nanoplatforms have been designed to induce apoptosis (*via* chemotherapeutics) or ferroptosis (*via* GPX4 inhibitors or iron-based therapies), few integrate both pathways in a sequential, biomarker-driven manner. Our results demonstrate that NT not only triggers significant apoptosis (Fig. 5A) but also potently induces ferroptosis under hypoxia, a condition where apoptosis is often suppressed (Fig. 5E-H). This dual-pathway attack is mechanistically supported by the downregulation of GPX4 and depletion of GSH, hallmarks of ferroptosis, alongside Caspase-3 activation. This approach addresses a key limitation of monotherapies: adaptive resistance. For example, anti-angiogenic therapies (e.g., bevacizumab) often exacerbate hypoxia, leading to HIF-1α-mediated survival and invasion [53]. NT provides a complementary kill switch by simultaneously activating ferroptosis, a death pathway that is not affected by typical apoptotic resistance mechanisms. This synergy is reflected in the profound tumor growth inhibition in vivo (Fig. 7C-E), significantly outperforming single-agent treatments (DHA or THP alone). The dual death signature was visually confirmed *via* Bio-TEM (Fig. S22), showcasing both apoptotic nuclear condensation and ferroptotic mitochondrial shrinkage within the same tumor, a compelling demonstration of coordinated cytotoxicity.

Our integrated omics analysis provides a systems-level validation of NT’s multi-targeted mechanism. The transcriptomic data reveal significant enrichment in p53 signaling, apoptosis, lipid metabolism, and angiogenesis pathways (Fig. 8C-D). This suggests that NT’s action converges on p53, a master regulator that can simultaneously promote apoptosis (PUMA/BAX) and ferroptosis (SLC7A11). This p53-mediated dual death induction represents a sophisticated biological outcome rarely achieved by single drugs. The metabolomic findings further highlight ROS as the central node orchestrating NT’s effects (Fig. 8E-F). The significant enrichment of the “Chemical carcinogenesis – ROS” pathway connects the upstream ROS burst (from DHA and disrupted mitochondria) to downstream execution of both apoptosis and ferroptosis, as well as anti-angiogenesis. This systems-level insight aligns with and extends recent approaches in cancer therapy, where modulating central metabolic or redox nodes is seen as more effective than targeting single proteins.

However, despite these promising results, certain limitations exist. While the biocompatibility and safety of NT were confirmed through in vitro and in vivo biosafety evaluations, the long-term toxicity and systemic effects of NT require further investigation. Additionally, the use of mouse models, although informative, may not fully capture the heterogeneity and complexity of human PDAC. The in vivo experiments in this study utilized a relatively small sample size, which limits the statistical robustness of the findings. To address this, future studies will increase the sample size to improve statistical power and reproducibility. Moreover, while this study demonstrates NT’s efficacy in tumor targeting and vascular disruption, direct comparisons with clinically relevant PDAC treatments, such as gemcitabine or FOLFIRINOX, were not conducted. A critical next step will be to compare NT with these standard treatments to assess its relative advantages and clinical potential. Finally, the modular design of NT allows for the incorporation of other targeting ligands or therapeutic cargos, making it a versatile platform for treating other solid tumors with dense stroma and oxidative stress.

## Conclusions

In this study, we developed a Venus flytrap-inspired nanoflytrap (NT) that integrates tumor-targeting specificity, deep stromal penetration, and dual apoptosis-ferroptosis induction for PDAC therapy. NT was successfully synthesized and characterized, with optimal physicochemical properties, including ROS-responsive drug release and MRI contrast capabilities. The NT exhibited exceptional tumor-targeting efficiency through p32 receptor binding and achieved deep penetration into dense PDAC spheroids. Mechanistically, NT synergistically induced mitochondrial-mediated apoptosis and DHA-driven ferroptosis, overcoming hypoxia-induced resistance. In vivo studies confirmed enhanced tumor accumulation, significant antitumor efficacy, and excellent biocompatibility. Through integrated transcriptomic and metabolomic analyses, NT treatment was found to coordinately activate apoptosis, disrupt lipid metabolism, and inhibit tumor angiogenesis, largely driven by ROS accumulation. NT achieves its potent antitumor effect by concurrently disrupting multiple cell death and vascular support pathways. These findings highlight NT as a promising theranostic platform for PDAC.

## Supplementary Information

Supplementary Material 1

Supplementary Material 2

## Data Availability

The data that support the findings of this study are available from the corresponding author upon reasonable request.
