# Supplementary material for: Activatable nanoflytrap achieves synergistic “Capture-Block-Regulate” therapy of pancreatic ductal adenocarcinoma
Source: J Nanobiotechnology. 2026 Apr 21;24:534. doi: 10.1186/s12951-026-04420-3 (PMC13244865; doi:10.1186/s12951-026-04420-3)
Supplement: Supplementary file 2 — Supplementary Material 2 [file 12951_2026_4420_MOESM2_ESM.docx]

**Activatable Nanoflytrap Achieves Synergistic “Capture-Block-Regulate” Therapy of Pancreatic Ductal Adenocarcinoma**

Kai Guo^a,b,†^, Shuai Ren^a,†^, Yingying Cao^a,†^, Huifeng Zhang^a^, Jin Cui^b^, Li Xian Yip^c^, Ximing Wang^b,^*, Ying Tian^a,^*, Lina Song^a,^*, Zhongqiu Wang^a,^*

*^a^Department of Radiology, Affiliated Hospital of Nanjing University of Chinese Medicine, Nanjing, 210029, China*

*^b^Department of Radiology, Shandong Key Laboratory of Multimodal Imaging Artificial Intelligence and Genetics, Shandong Provincial Hospital Affiliated to Shandong First Medical University, Jinan 250021, China*

*^C^Department of Chemical and Biomolecular Engineering, National University of Singapore, Singapore 117585, Singapore*

^†^These authors contributed equally

**Corresponding author*

*E-mail address:* zhongqiuwang@njucm.edu.cn (Z. Wang), songlina@njucm.edu.cn (L. Song),

ty52111@sina.com (Y. Tian), wxming369@163.com (X. Wang)

**Experimental Section**

**Synthesis of NT**

Initially, 100 mg of PLGA_10k_-TK-PEI_10k_ was dissolved in 300 μL of methanol under ultrasonication, followed by the addition of 2.5 mL of chloroform and thorough mixing. DHA and Span emulsifier were then incorporated into the solution with further ultrasonication to ensure complete dissolution, forming the oil phase. For the emulsion preparation, the inner aqueous phase (200 µL of gadopentetate meglumine solution) was first emulsified with the oil phase by vortexing and sonication (40% power, 3 min) to form a W1/O emulsion. This primary emulsion was then transferred into a PVA solution (1%, 10 mL) serving as the outer aqueous phase, followed by a second cycle of vortexing and sonication (40% power, 4 min) to obtain the final W1/O/W2 double emulsion. After overnight magnetic stirring (100 rpm), the emulsion was purified *via* 100 kDa ultrafiltration with five sterile water washes, yielding DHA- and gadolinium-loaded PLGA nanoparticles (DHA-PTP) in a final volume of 10 mL. For surface functionalization, 50 mg of PAA-adsorbed DHA-PTP was ultrafiltered with 100 mM MES buffer (pH 4.8), resuspended in 4 mL of buffer, and then reacted with 4 mg of THP peptide (CGKRK- _D_(KLAKLAK)_2_) and 50 mg of EDC in a total volume of 5 mL under overnight shaking (37°C). The resulting NT nanoparticles were obtained after ultrafiltration to remove unbound THP and final resuspension in 5 mL of purified water. As a hydrophobic molecule, FITC was incorporated into NT using the W1/O/W2 double emulsion method. In this approach, FITC was dissolved in the oil phase together with PLGA-TK and DHA, ensuring that FITC remains encapsulated within the hydrophobic core of the nanoparticles. The oil phase was then emulsified in the aqueous phase to form stable NT nanoparticles. Regarding the impact on NT properties, we have carefully assessed the potential effects of FITC labeling on the size and surface charge of NT. Our results show that FITC incorporation does not significantly alter the core properties of NT (Fig. S39). This suggests that the FITC labeling strategy does not interfere with the integrity or function of NT, thus enabling reliable monitoring of cellular uptake without compromising the nanoparticle's therapeutic properties.

**Bioinformatics Analysis**

A subset of pancreatic cancer samples with matched clinical data was retrieved from the TCGA PAAD database, while healthy pancreatic tissue data was sourced from the GTEx database. The study cohort comprised 179 pancreatic cancer samples from TCGA and 171 normal pancreatic tissue samples from GTEx for comparative analysis. RNA-seq data (in TPM format) from both databases were uniformly processed using the Toil pipeline via the UCSC XENA platform (https://xenabrowser.net/datapages/). Data visualization was performed using the ggplot2 package in R.

**Molecular Docking**

We constructed 3D models of PLL and ICG using Chimera and performed protein-peptide docking with AutoDock 4.2. The pre-docking preparation included: 1) adding polar hydrogen atoms, 2) assigning partial atomic charges *via* Gasteiger calculations, and 3) defining a docking box encompassing the entire protein structure. Docking simulations employed the Lamarckian Genetic Algorithm with default software parameters. The resultant complexes were visualized using PyMOL and LIGPLOT.

**Three-Dimensional tumor cell spheroids**

Agarose (0.16 g) was dissolved in 10 mL of DMEM medium by heating in a 100°C water bath. The solution was rapidly transferred to a 96-well plate and allowed to solidify at room temperature. Subsequently, MiaPaCa-2 cells suspended in Matrigel were seeded onto the agarose-coated plate. Spheroids were cultured in a 300 µm-diameter microwell plate under standard incubator conditions. For uptake studies, spheroids were incubated with FITC-labeled NT solution. Prior to confocal imaging, the culture medium containing FITC-NT was aspirated, and the spheroids were washed three times with PBS to remove unbound or superficially adsorbed nanoparticles. Z-stack images were acquired using confocal microscopy, and fluorescence intensity across different depth layers was quantified with ImageJ software.

**Cytotoxicity Assessment**

Log-phase MiaPaCa-2, BxPC-3, HUVEC, and HPNE cells were trypsinized and seeded in 96-well plates at 8000 cells/well. PBS was added to peripheral wells to minimize evaporation. The cells were cultured at 37°C with 5% CO_2_. The following solutions were prepared in concentration gradients (0-200 μg/mL for NT and PLGA-TK-PEI, 0-400 μM for DHA, 0-40 μM for THP, 0-200 μg/mL for DHA-KLA), mixed thoroughly, and stored at 4°C. After the cells attached, the medium was replaced with drug solutions. The controls included cell-only and drug-only wells. Parallel experiments were conducted under hypoxia (95% N_2_, 5% CO_2_, 37°C). After a 24-hour incubation, 20 μL of CCK-8 was added per well. The absorbance (450 nm) was measured after 1 hour. The cell viability was calculated as: (ODtreatment - ODcell-only) / (ODmedium - ODcell-only) × 100%. Additionally, MiaPaCa-2 and HUVEC cells were treated with DHA (100 μM), THP (10 μM), DHA-KLA (100 μg/mL), or NT (100 μg/mL). After 24 hours, the cells were washed with PBS and stained with Calcein-AM/PI (0.5 μL each in 1 mL of PBS) for 30 minutes. The fluorescence was imaged (Ex/Em: 494/517 nm for live cells, 535/617 nm for dead cells).

**Biodistribution Imaging**

To evaluate the tumor targeting efficacy, a subcutaneous pancreatic cancer model was established by subcutaneous injection of MiaPaCa-2 cells in nude mice. When the tumors reached approximately 100 mm^3^, the mice were randomly divided into two groups and intravenously administered with either IR780-labeled DHA-KLA or IR780-labeled NT. The real-time biodistribution was monitored at predetermined time points (0, 1, 6, 12, 24, and 48 h post-injection) using an IVIS Spectrum imaging system with excitation/emission settings of 745/800 nm.

**MRI Experiment**

For *in vivo* MR imaging studies, MiaPaCa-2 tumor-bearing nude mice were anesthetized using isoflurane (4% for induction and 2% for maintenance in 0.5 L/min oxygen) and randomly assigned to receive intravenous injections of either DHA-KLA or NT. MRI was performed using a clinical 3.0 Tesla scanner (Siemens Magnetom Trio) at pre-injection baseline and post-injection time points (1, 6, 12, 24, and 48 h). Imaging parameters were optimized for tumor detection: TR = 482 ms, TE = 13 ms, flip angle = 130°, FOV = 60 mm, matrix size = 192×192, slice thickness = 0.8 mm.

***In Vivo* Antitumor Studies**

Tumor volume was calculated using the formula: V = (length × width^2^) / 2. MiaPaCa-2 cells were subcutaneously injected into the right hind limb of nude mice to establish xenograft tumors. Upon reaching a tumor volume of ~100 mm^3^, animals were randomly assigned to five experimental cohorts: Saline, DHA, THP, DHA-KLA, and NT. For all *in vivo* experiments, the number of animals per group was n = 3. The animals were randomly assigned to experimental groups using a computer-generated random number table to avoid selection bias. Measurements and data analysis were performed in a blinded manner. After data collection, group allocations were revealed through numerical coding to ensure objectivity. Treatments were administered *via* tail vein injection. Body weight and tumor dimensions were monitored throughout the 15-day study period. At the endpoint, mice were euthanized, tumors were excised, and final tumor weight and volume were measured for comparative analysis.

**Hemolysis Analysis**

For hemolysis analysis, whole blood collected from mice was subjected to sequential centrifugation (300 × g, 10 min, 4°C) and PBS washes to isolate erythrocytes. The pelleted red blood cells (RBCs) were resuspended in PBS and incubated with serial concentrations of NT or DHA-KLA for 4 h at 37°C. After centrifugation, the supernatant was photographed to visually assess hemoglobin release. Quantitative hemolysis was determined spectrophotometrically by measuring absorbance at 540 nm, with water and saline serving as positive and negative controls, respectively.

**Data Analysis**

All data are presented as the mean from at least three independent experiments. Statistical comparisons between two groups were performed using Student’s t-test, while differences among multiple groups were analyzed by a one-way analysis of variance (ANOVA). A *p-value* of less than 0.05 was considered statistically significant.

**Supplementary Figures and Tables**

**
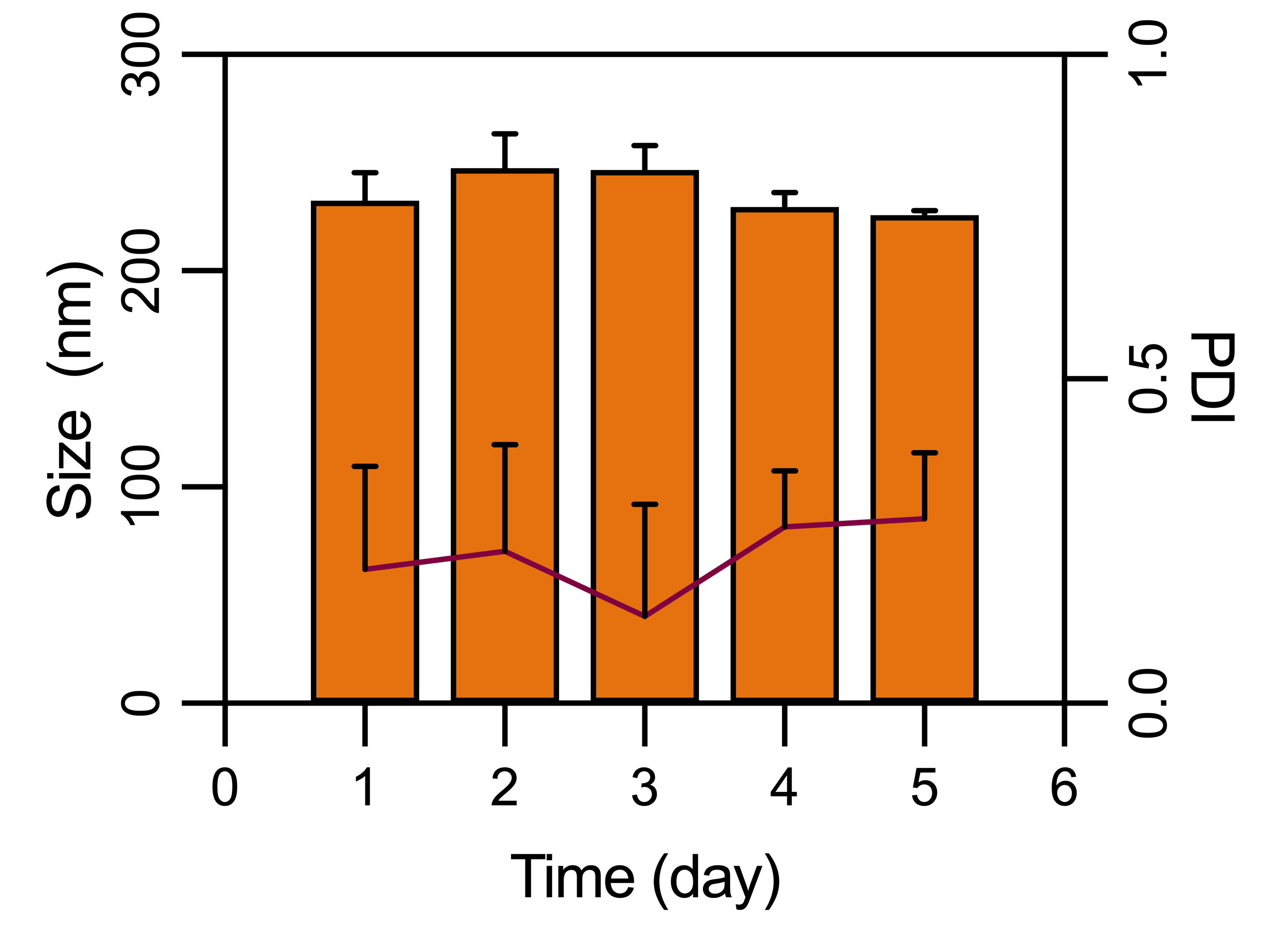
**

**Fig. S1** Stability analysis of particle size and PDI of NT.

**
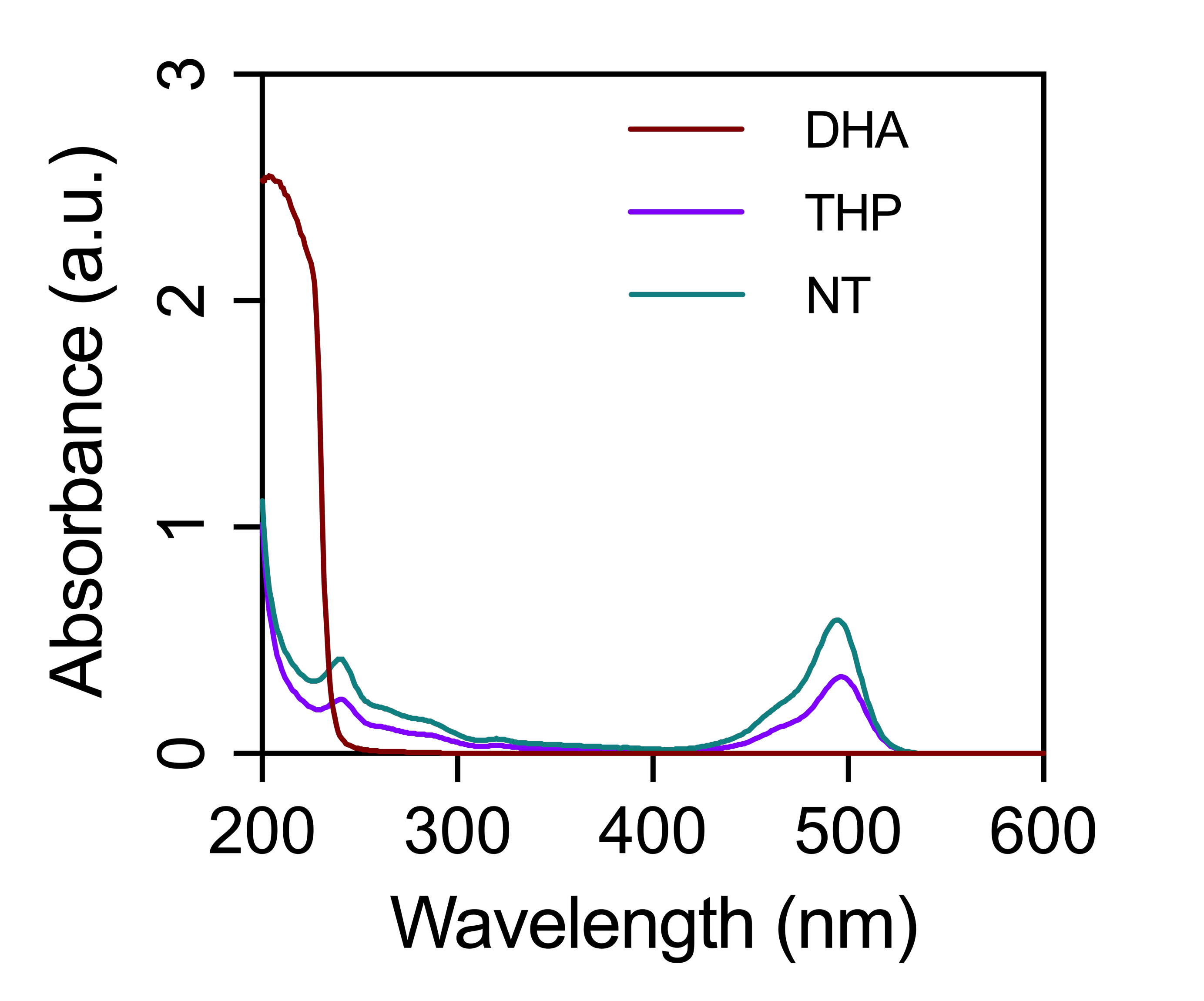
**

**Fig. S2** UV-Vis absorption spectra of DHA, THP, and NT.

**
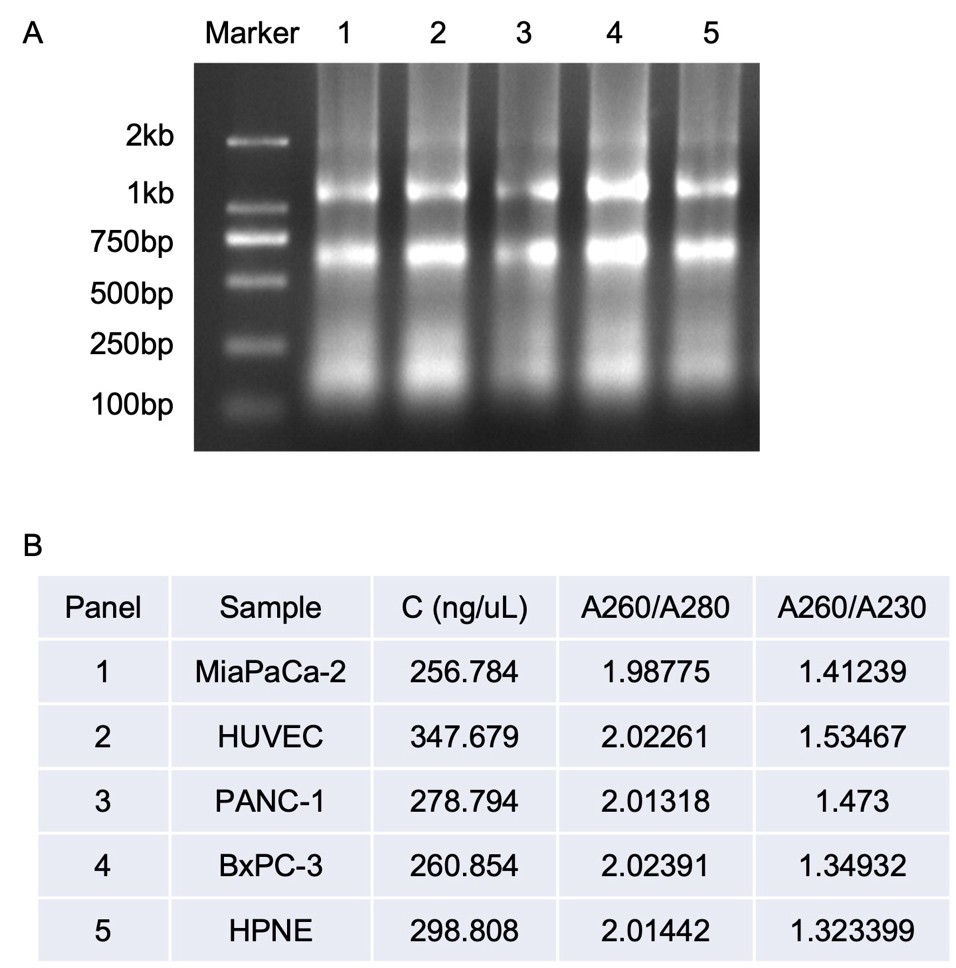
**

**Fig. S3** (**A**) Image of the electrophoresis gel for 1) MiaPaCa-2, 2) HUVEC, 3) PANC-1, 4) BxPC-3 and 5) HPNE cell line RNA samples. (**B**) Table with sample name, RNA concentrations (ng/μL), indicator of purity (ratio A260/A280), and indicator of purity (ratio A260/A230).


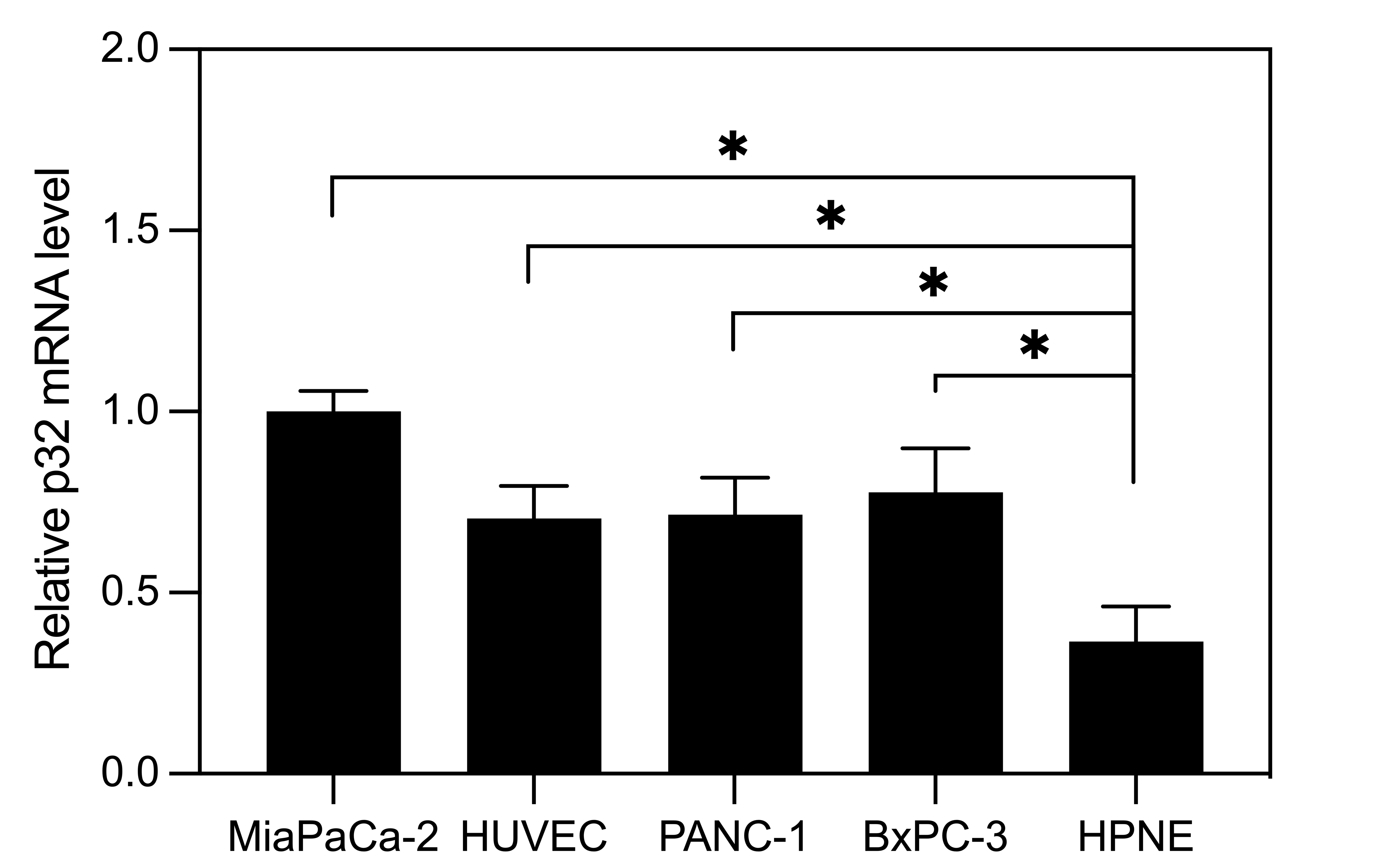


**Fig. S4** Q-PCR results showed the expression of p32 mRNA in MiaPaCa-2, HUVEC, PANC-1, BxPC-3 and HPNE cells.


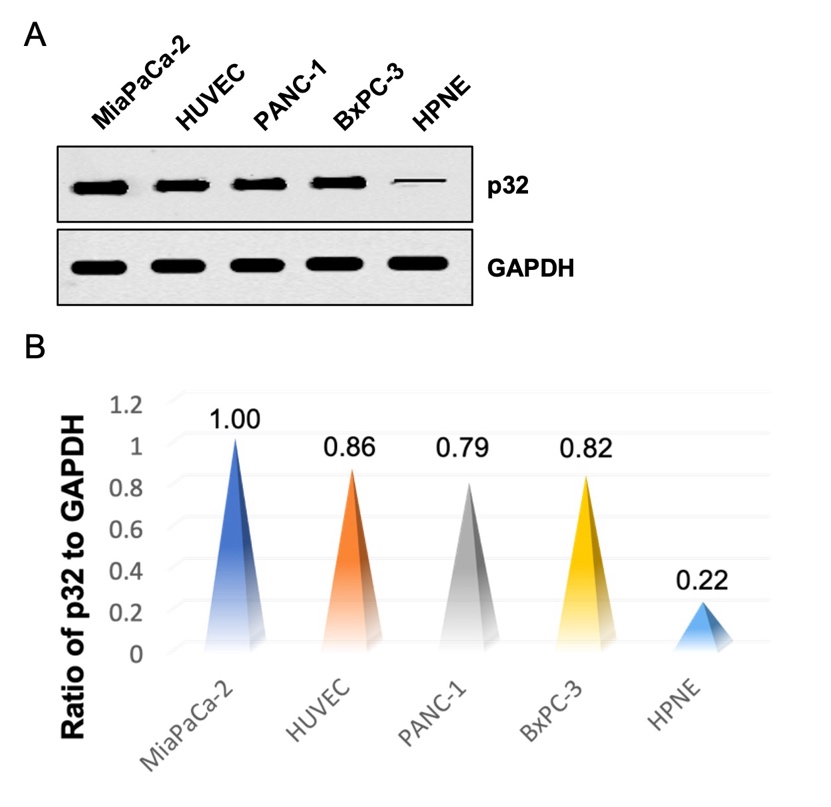


**Fig. S5** **A** Western blot analysis and (**B**) quantitative analysis of p32 protein expression in MiaPaCa-2, HUVEC, PANC-1, BxPC-3, and HPNE cell lines.


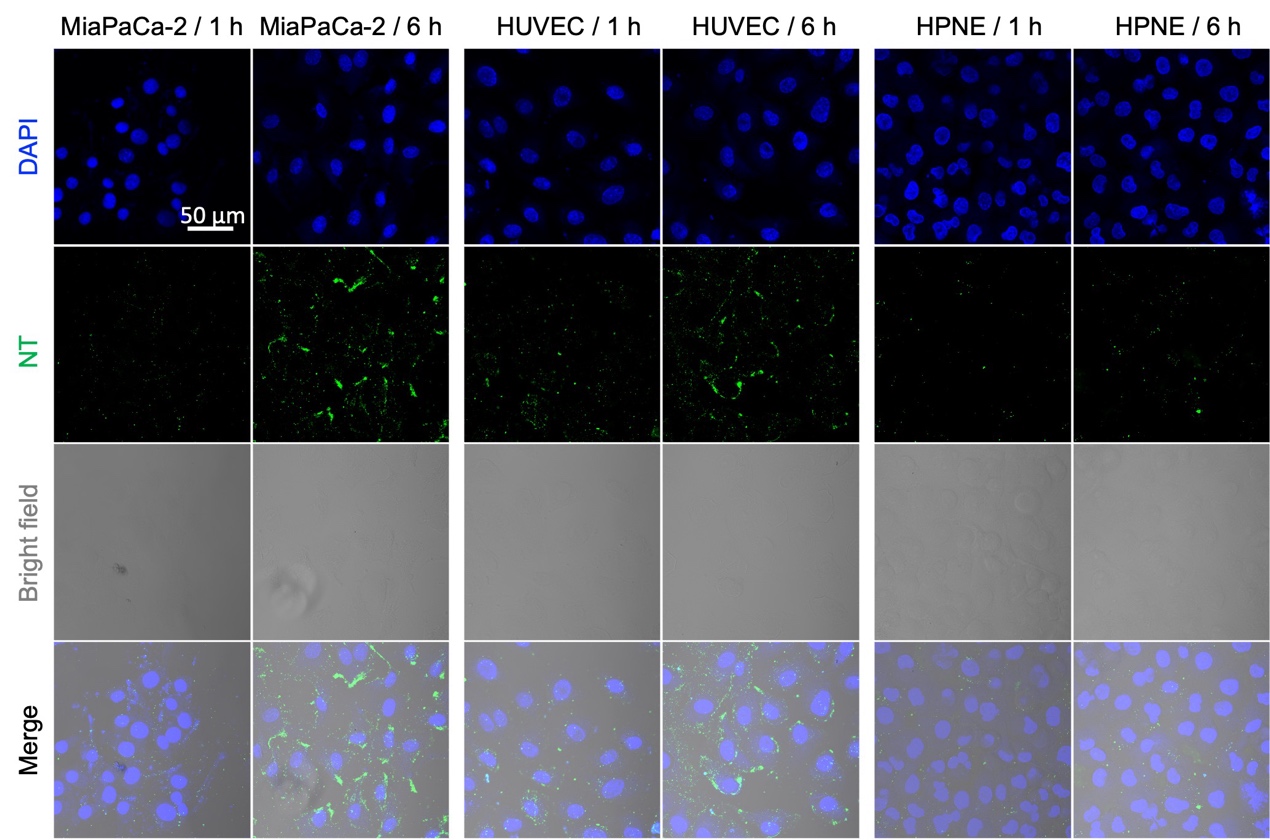


**Fig. S6** Confocal fluorescence imaging tracked NT internalization dynamics across three cell types (MiaPaCa-2, HUVEC, HPNE) following 1 h and 6 h exposures.


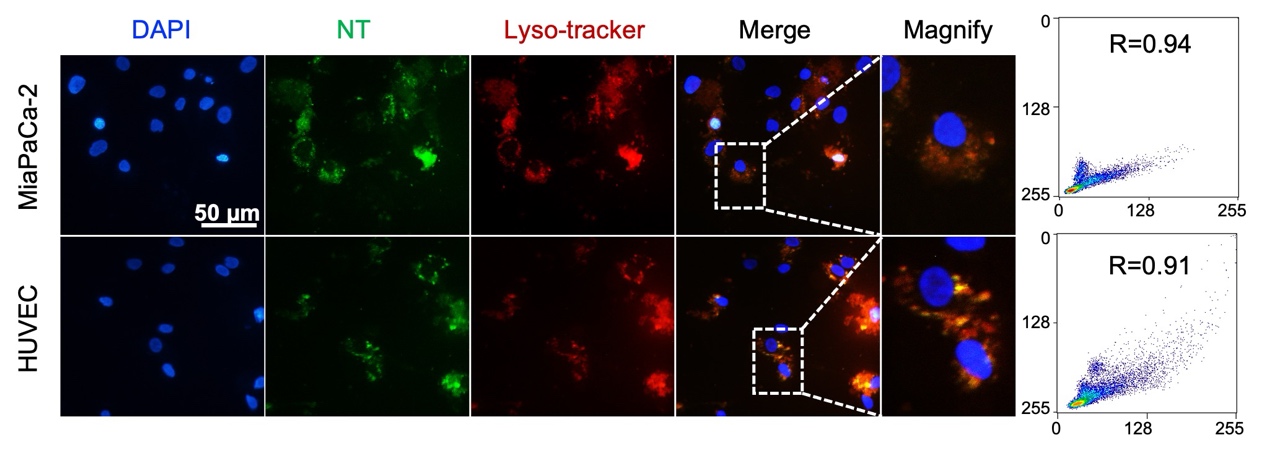


**Fig. S7** Lysosomal co-localization of NT in MiaPaCa-2 and HUVEC cells after 6 h incubation, analyzed by *Pearson’s* correlation coefficient.


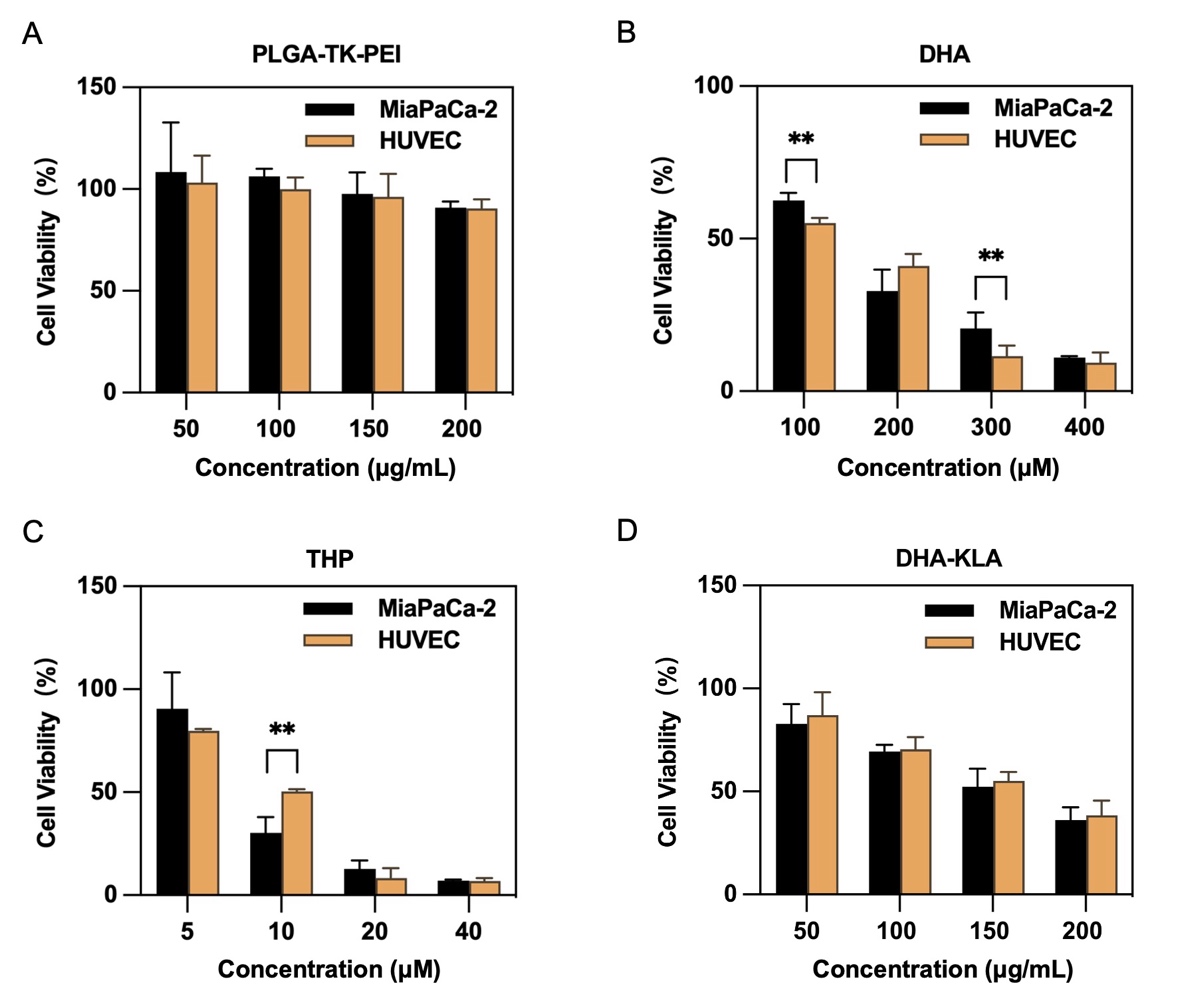


**Fig. S8** Effect on cell proliferation of MiaPaCa-2 and HUVEC at different concentrations of (**A**) PLGA-TK-PEI, (**B**) DHA, (**C**) THP, and (**D**) DHA-KLA. ***p*<0.01.


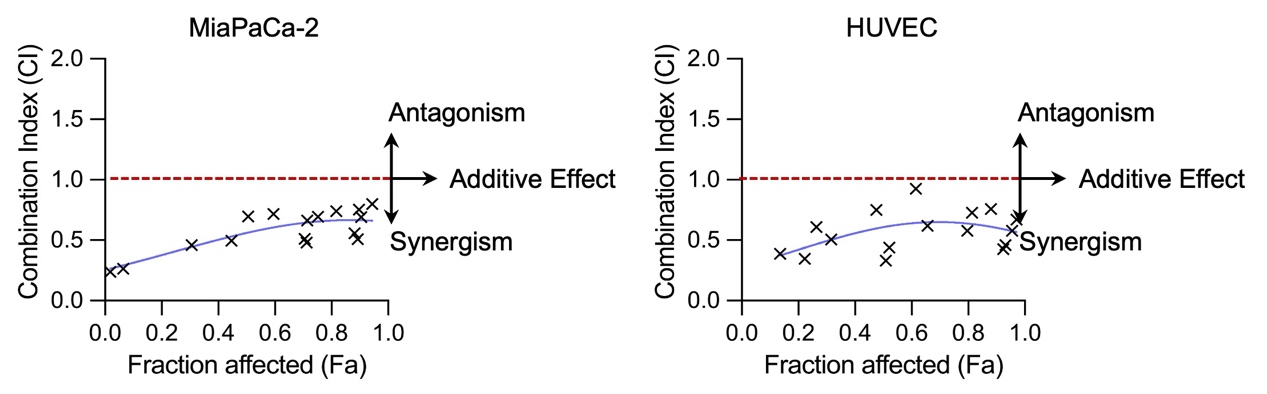


**Fig. S9** Calculation of the Chou-Talalay CI for the combination of DHA and THP against MiaPaCa-2 and HUVEC. CI < 1 indicates synergism.


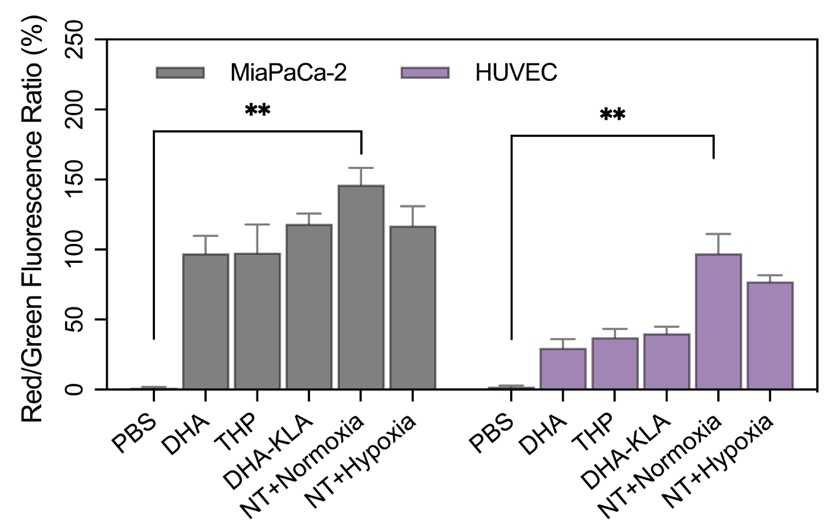


**Fig. S10** Quantitative analysis corresponding to the aforementioned treatments was performed. ***p*<0.01.


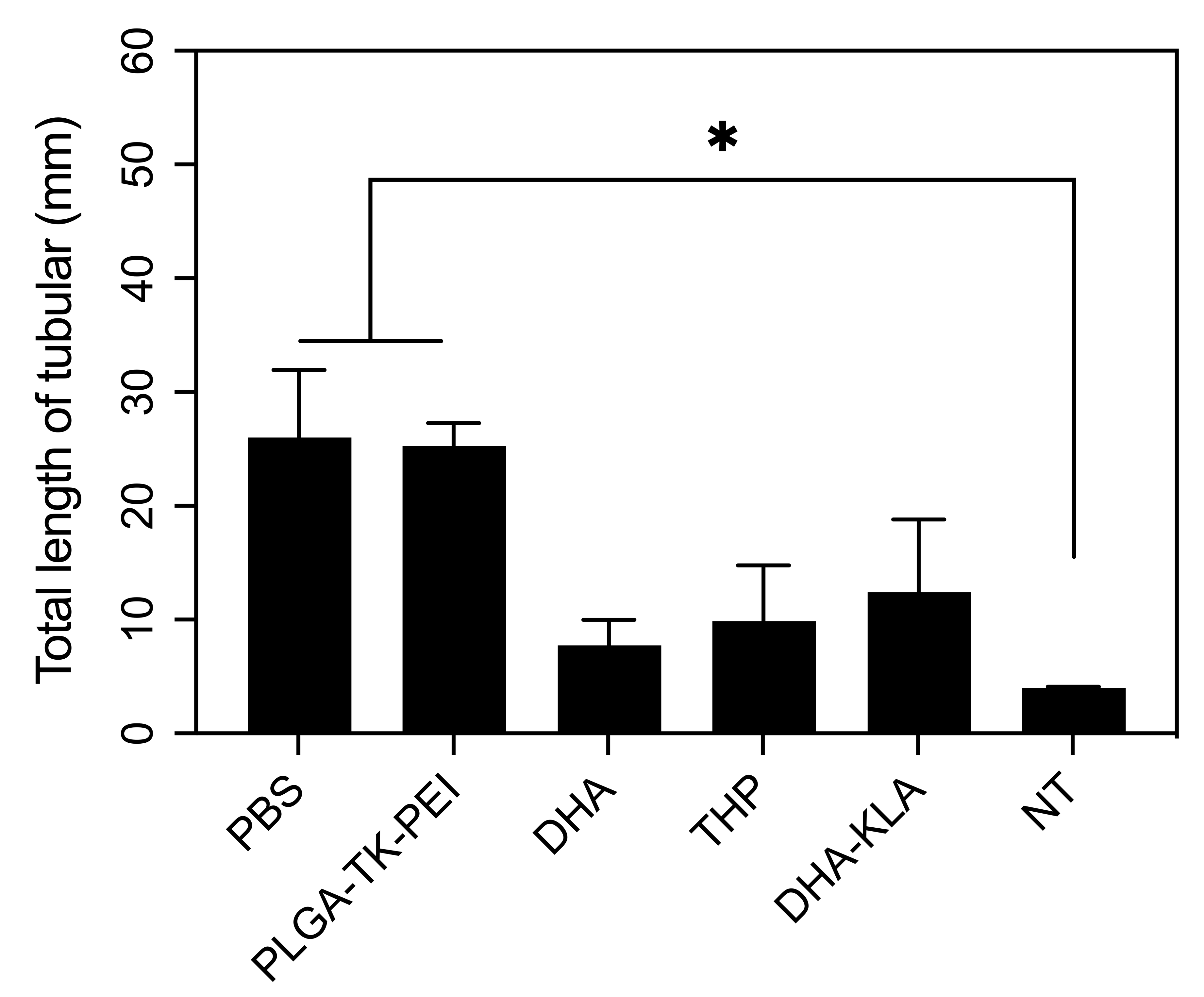


**Fig. S11** The statistical analysis of the tube formation assay. **p*<0.05.

**
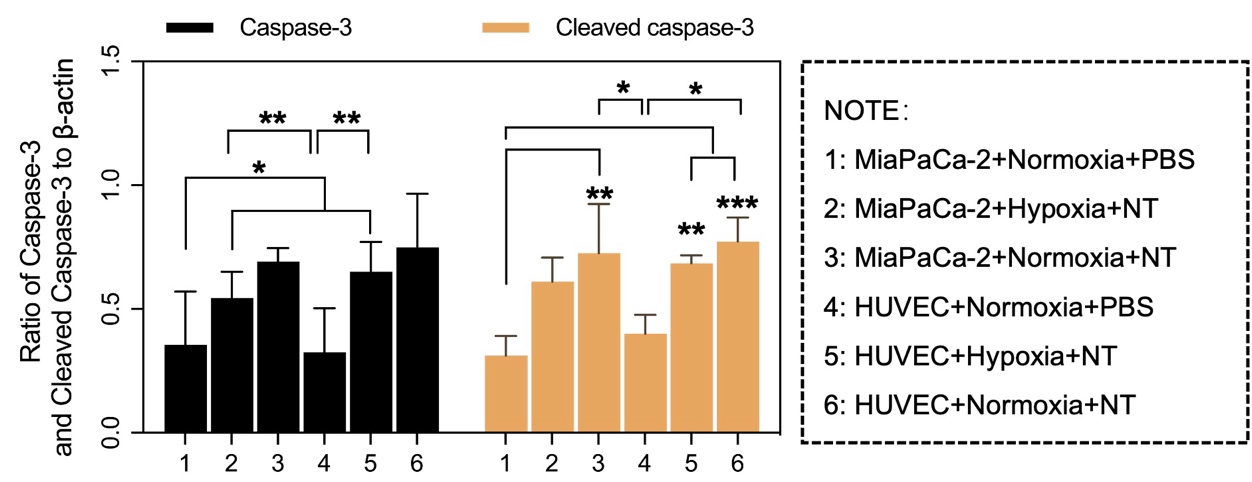
**

**Fig. S12** The Western blot was quantified and the relative expression of Caspase-3 and Cleaved Caspase-3 was calculated after normalizing with GAPDH. **p*<0.05, ***p*<0.01, ****p*<0.001.

**
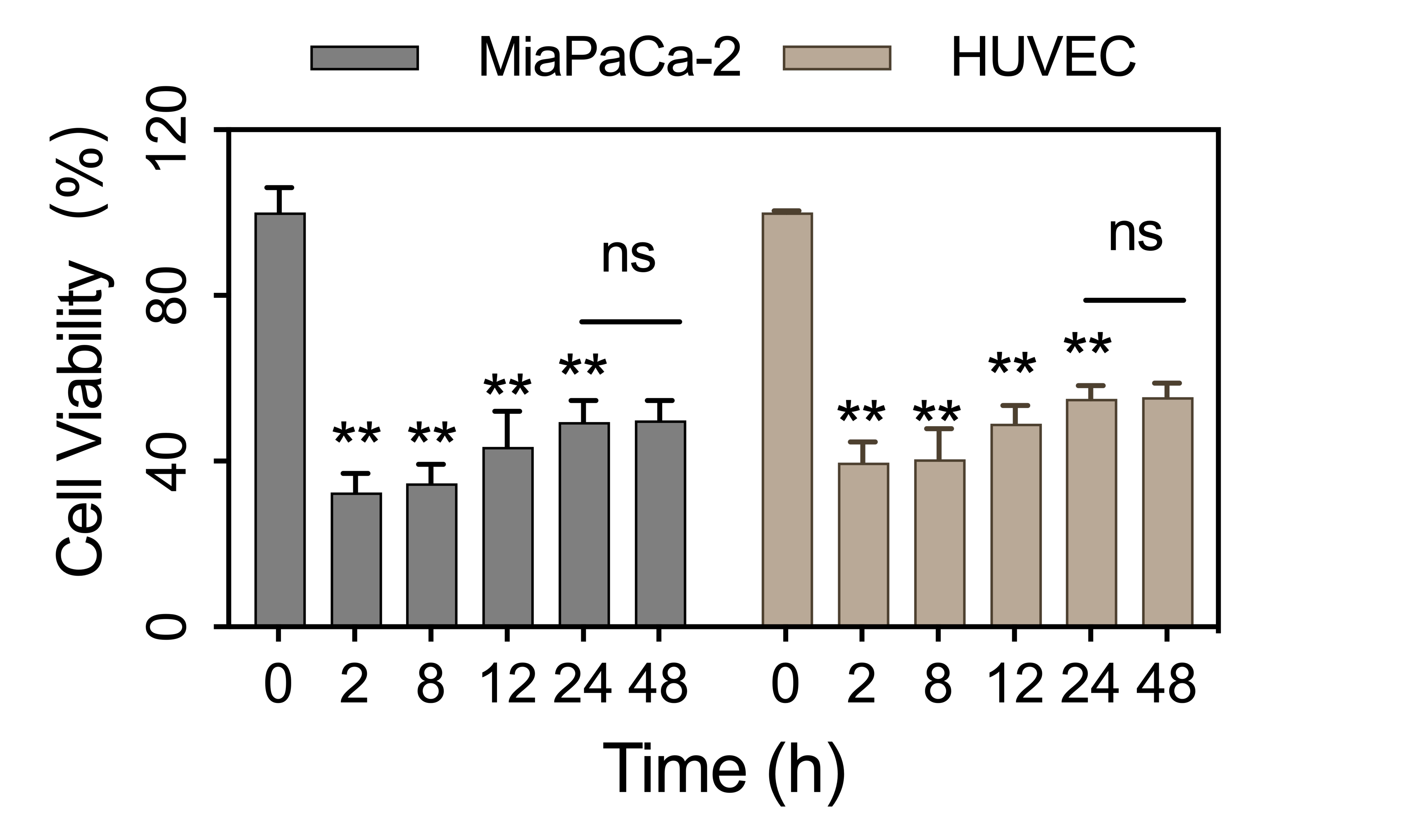
**

**Fig. S13** Time-dependent effect of Z-VAD-FMK on cell viability. Cells were pretreated with Z-VAD-FMK (40 μM) before being treated with NT alone for the indicated times. ***p*<0.01, ns>0.05.


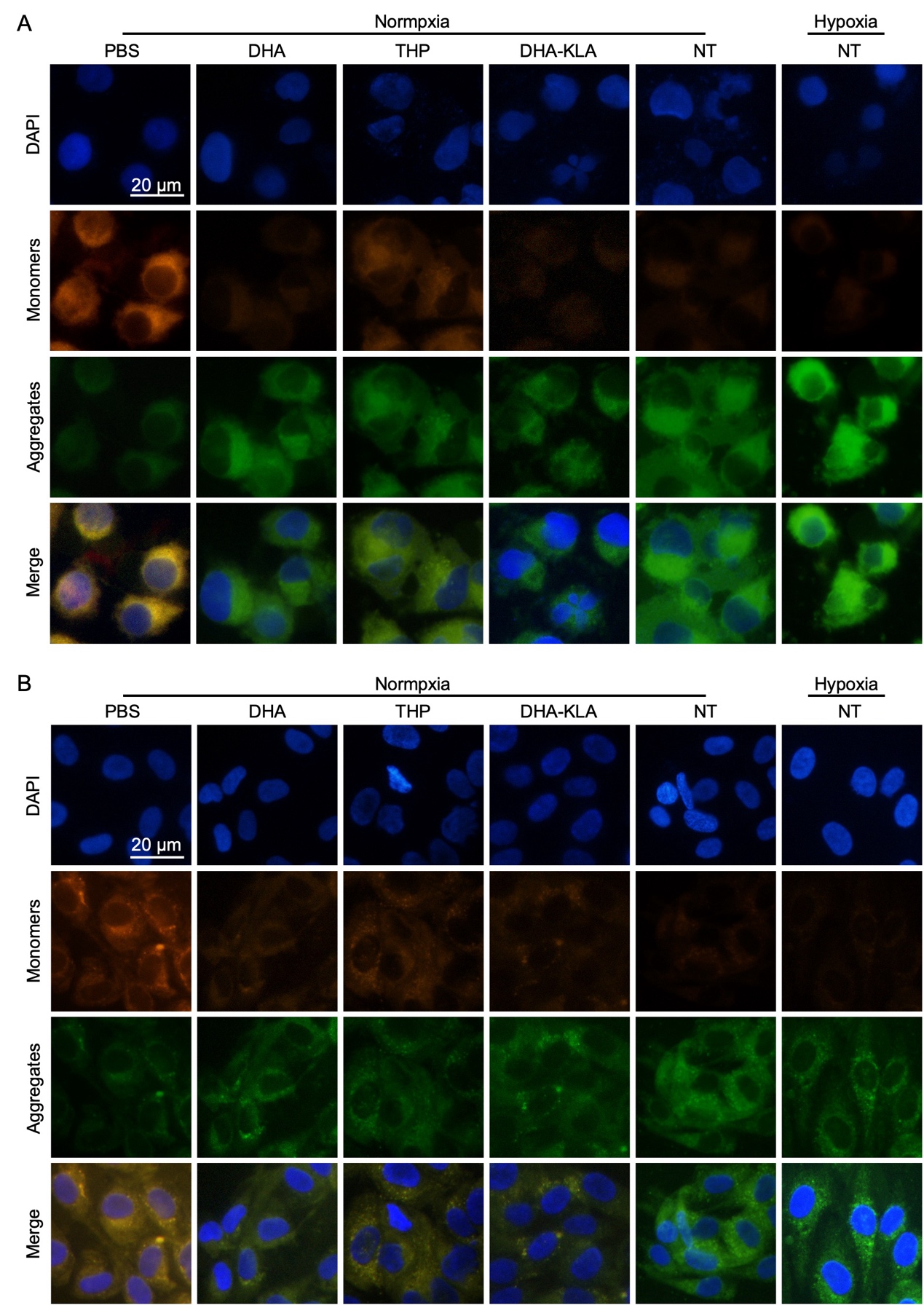


**Fig. S14** Fluorescence images of JC-1 assay to measure mitochondrial membrane depolarization in (**A**) MiaPaCa-2 and (**B**) HUVEC cells after different treatments. The red fluorescence emission corresponds to JC-1 aggregates (590 nm) formed in mitochondria with intact membrane potential (ΔΨm), while the green fluorescence (530 nm) indicates cytosolic JC-1 monomers resulting from ΔΨm dissipation.

**
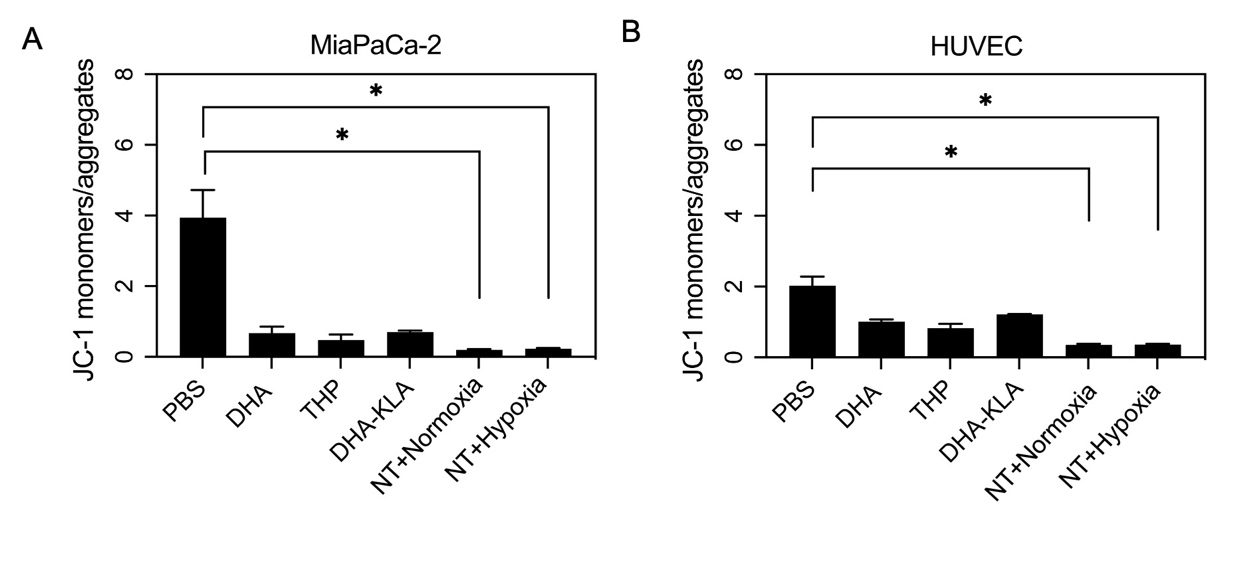
**

**Fig. S15** The ratio of monomeric JC-1 and aggregated JC-1 represented Δψm of (**A**) MiaPaCa-2 and (**B**) HUVEC cells after different treatments. **p*<0.05.


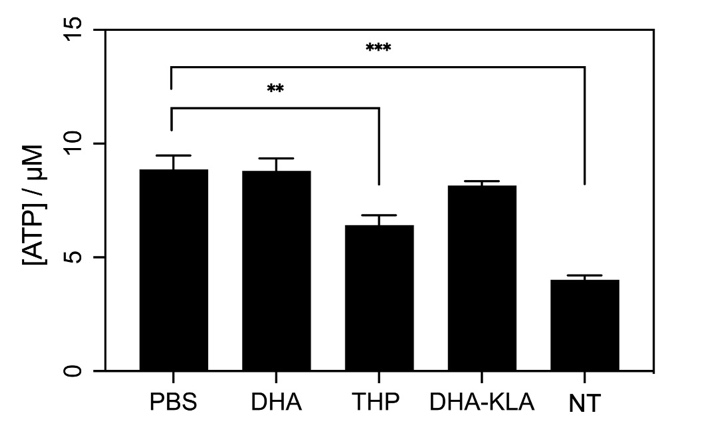


**Fig. S16** ATP levels in MiaPaCa-2 cells with various treatments. ***p*<0.01, ****p*<0.001.


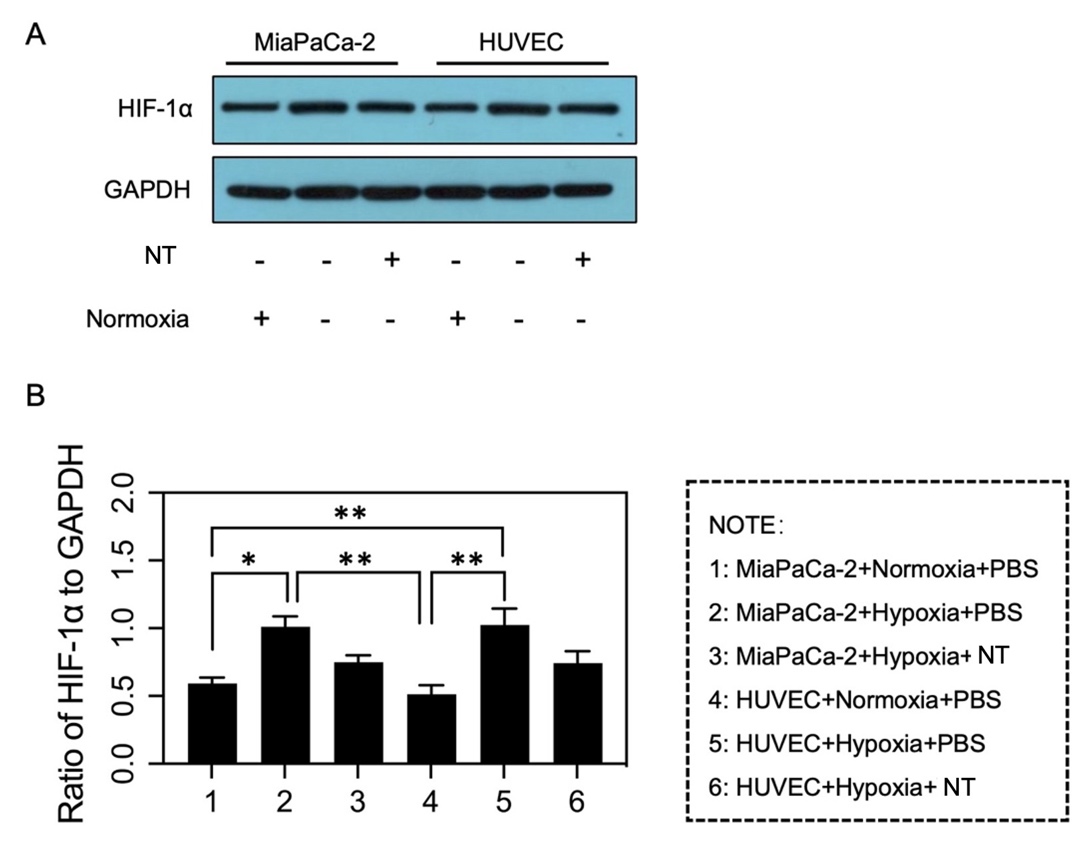


**Fig. S17 A** Western blot and (**B**) quantitative analysis of HIF-1α expression in MiaPaCa-2 and HUVEC cells with various treatments. **p*<0.05, ***p*<0.01.


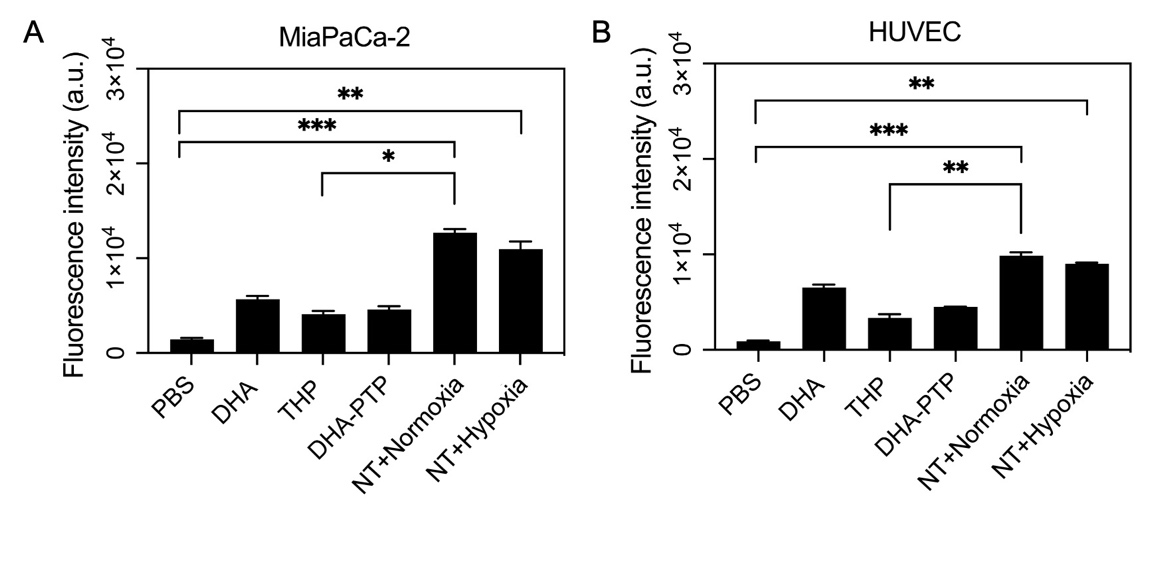


**Fig. S18** The quantitative analysis of ROS fluorescence intensity in MiaPaCa-2 and HUVEC cells after different treatments. **p*<0.05, ***p*<0.01, ****p*<0.001.

**
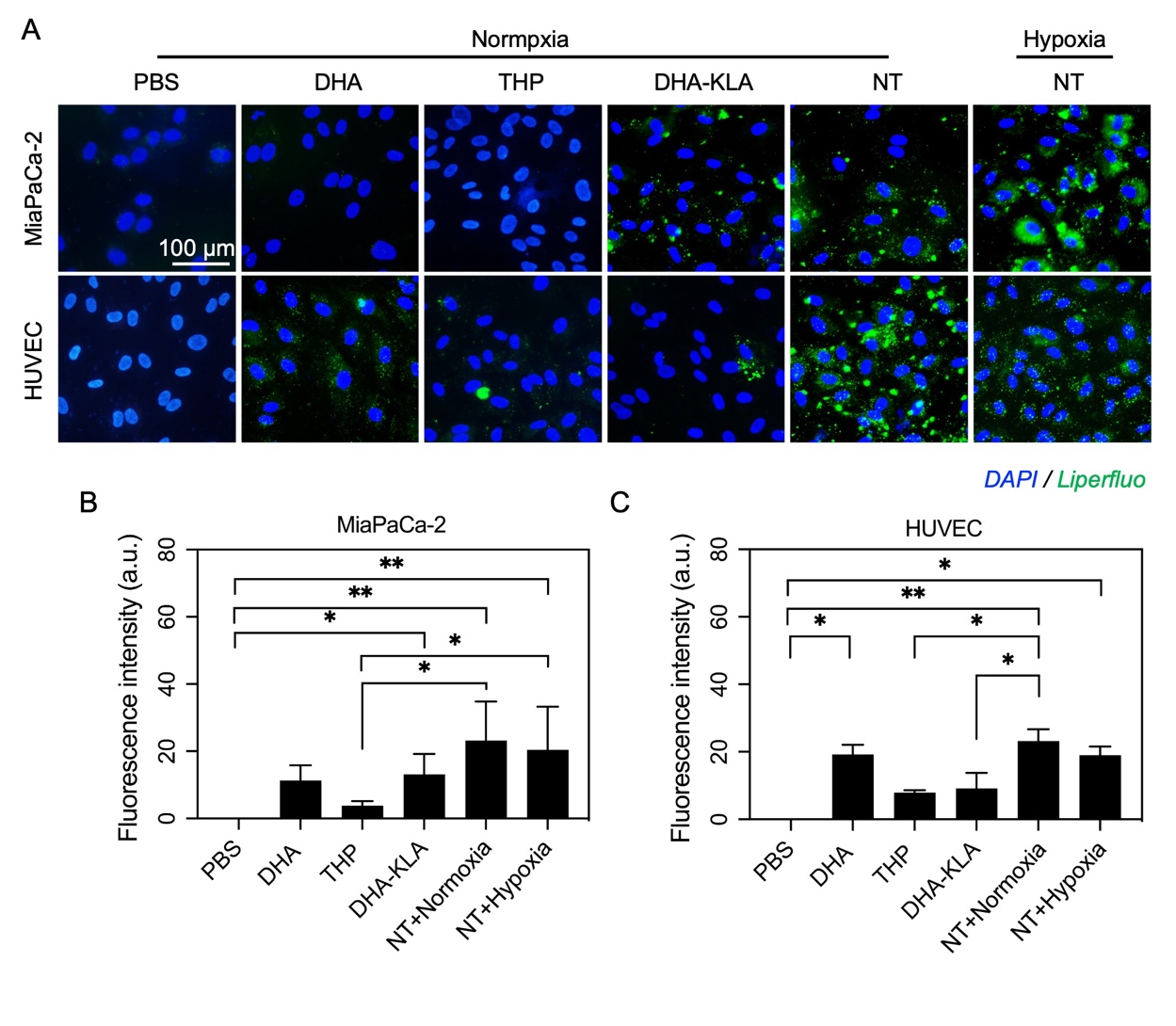
**

**Fig. S19** Fluorescence images of lipid peroxide in (**A**) MiaPaCa-2 and HUVEC cells with different treatments detected by liperfluo (green) for the lipid peroxide-specific oxidation. The quantitative analysis of Liperfluo fluorescence intensity in (**B**) MiaPaCa-2 and (C) HUVEC cells after different treatments. **p*<0.05, ***p*<0.01.


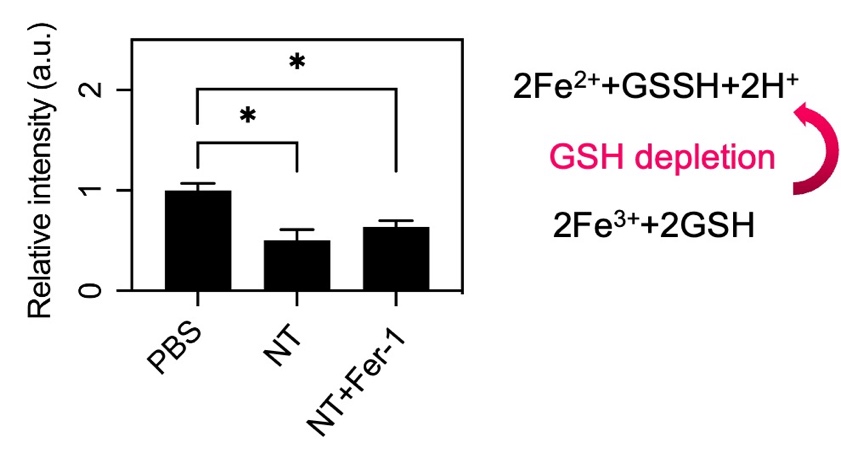


**Fig. S20** The relative intracellular GSH/GSSG values of MiaPaCa-2 cells (**p*<0.05).

**
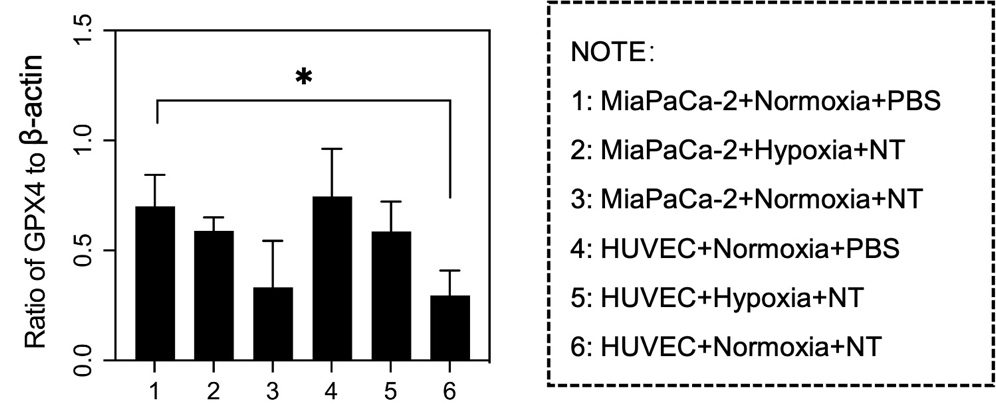
**

**Fig. S21** The quantitative analysis of GPX4 expression in MiaPaCa-2 and HUVEC cells with various treatments. **p*<0.05.


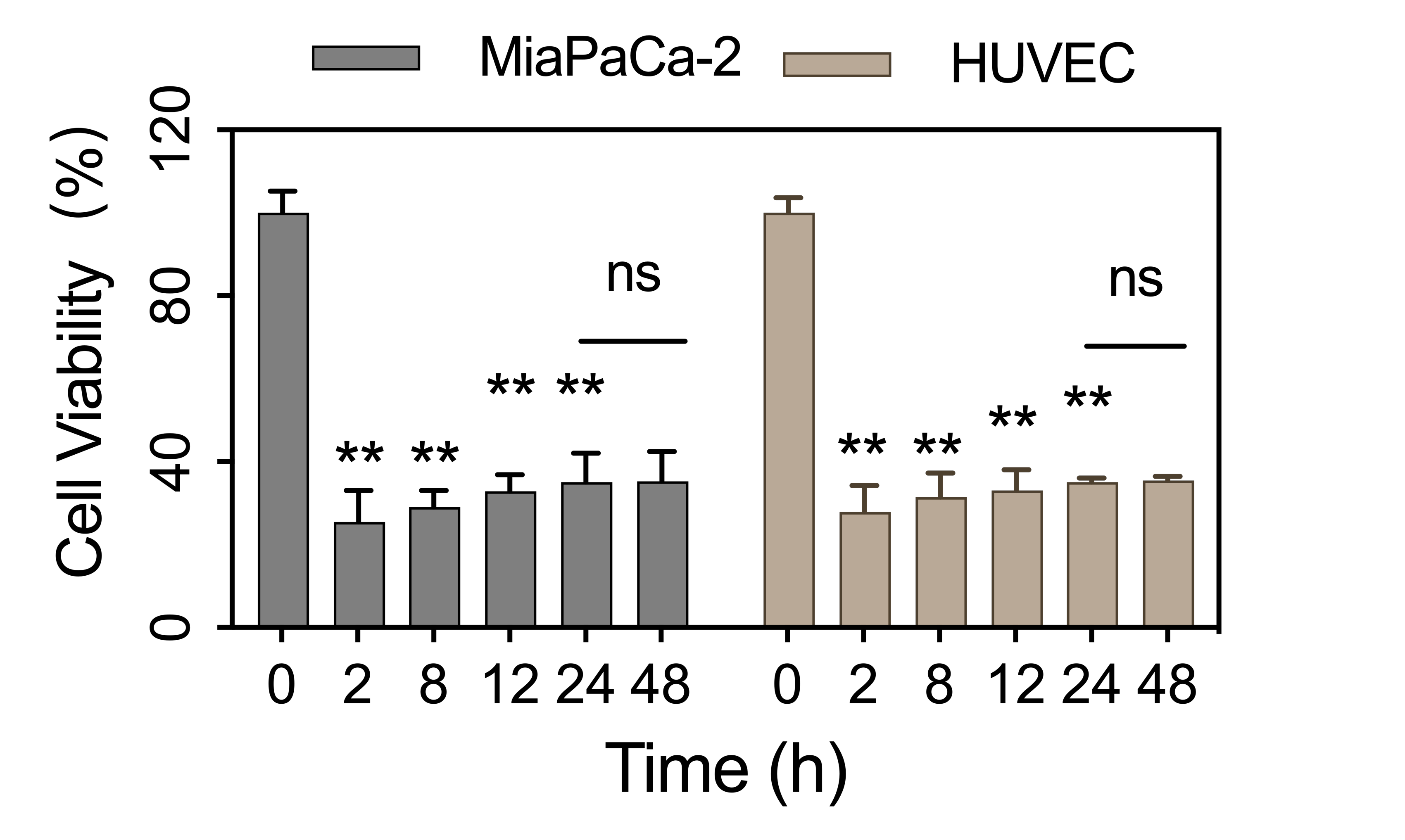


**Fig. S22** Time-dependent effect of Fer-1 on cell viability. Cells were pretreated with Fer-1 (200 nM) before being treated with NT alone for the indicated times.***p*<0.01, ns>0.05.


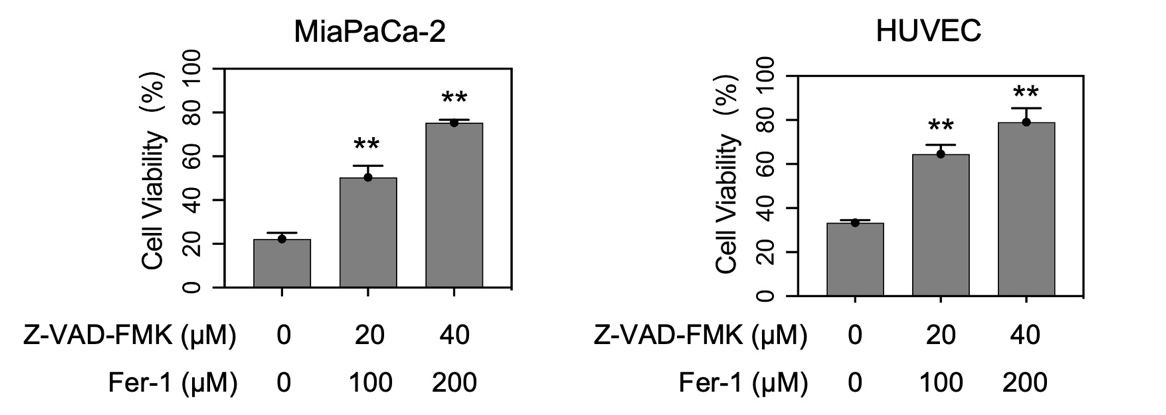


**Fig. S23** Dual-inhibitor analysis of NT-induced cytotoxicity in MiaPaCa-2 and HUVEC cells. Cells were treated with NT in the presence or absence of the pan-caspase inhibitor Z-VAD-FMK and the ferroptosis inhibitor ferrostatin-1 (Fer-1), and cell viability was measured after 24 h. Simultaneous inhibition of both pathways produced a markedly greater rescue effect (n = 5). ***p*<0.01.


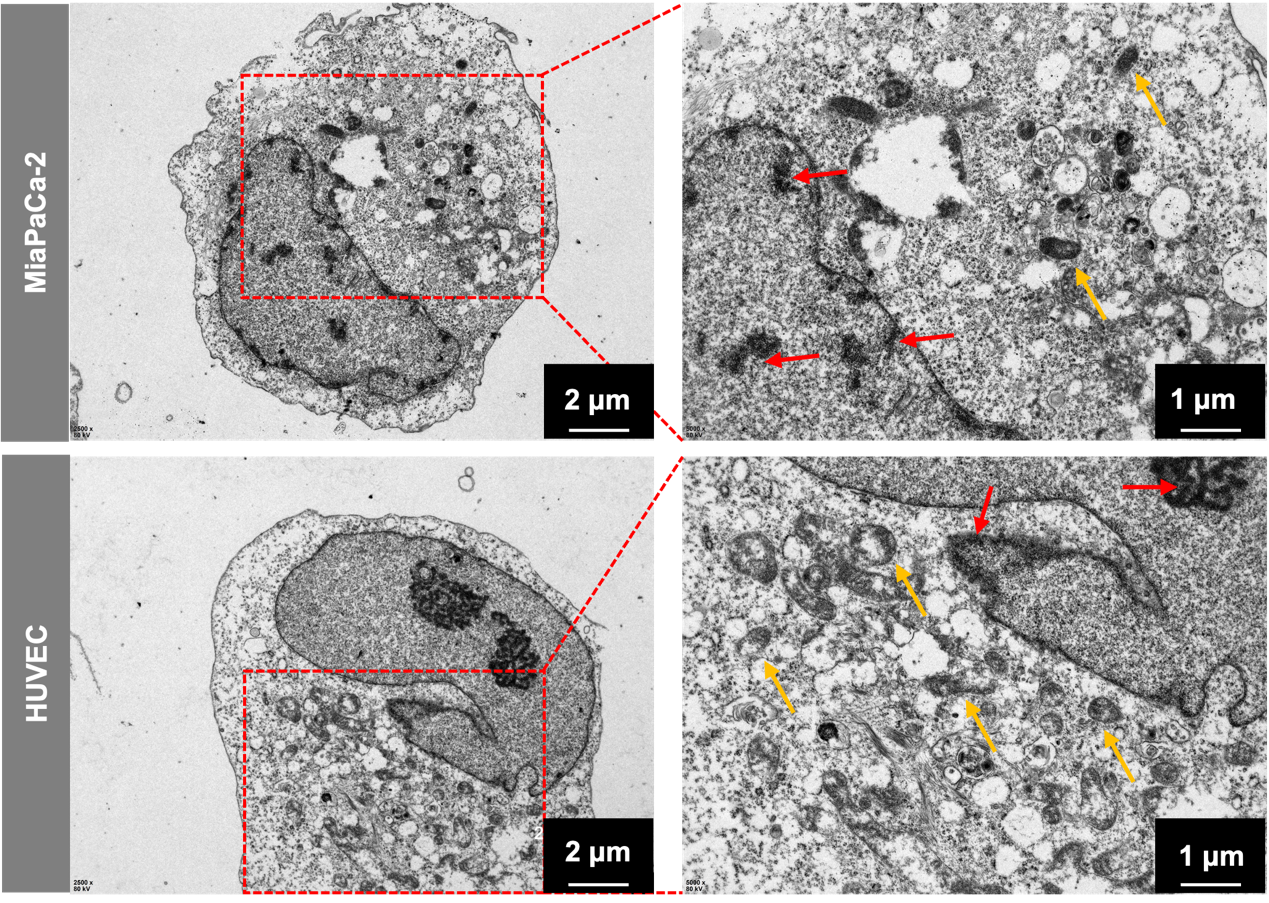


**Fig. S24** Ultrastructure of MiaPaCa-2 and HUVEC cells treated with NT (100 μg/mL, 24 h). Under bio-TEM, NT treatment induced mitochondrial pyknosis, mitochondrial fragmentation and the disappearance of mitochondrial ridge (yellow arrows). The nucleus had chromosome condensation and chromosome edge condensation (red arrows). Scale bar, 2 μm in the upper and lower left subpanels, and 1 μm in the upper and lower right subpanels.


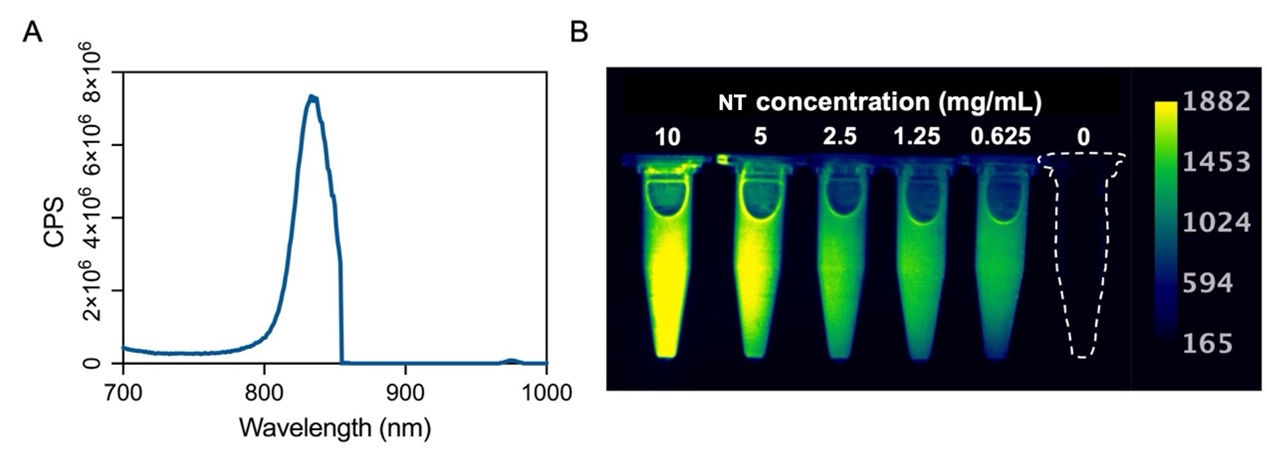


**Fig. S25** **A** Fluorescence excitation spectrum. (**B**) *In vitro* fluorescence imaging of NT at different concentrations.


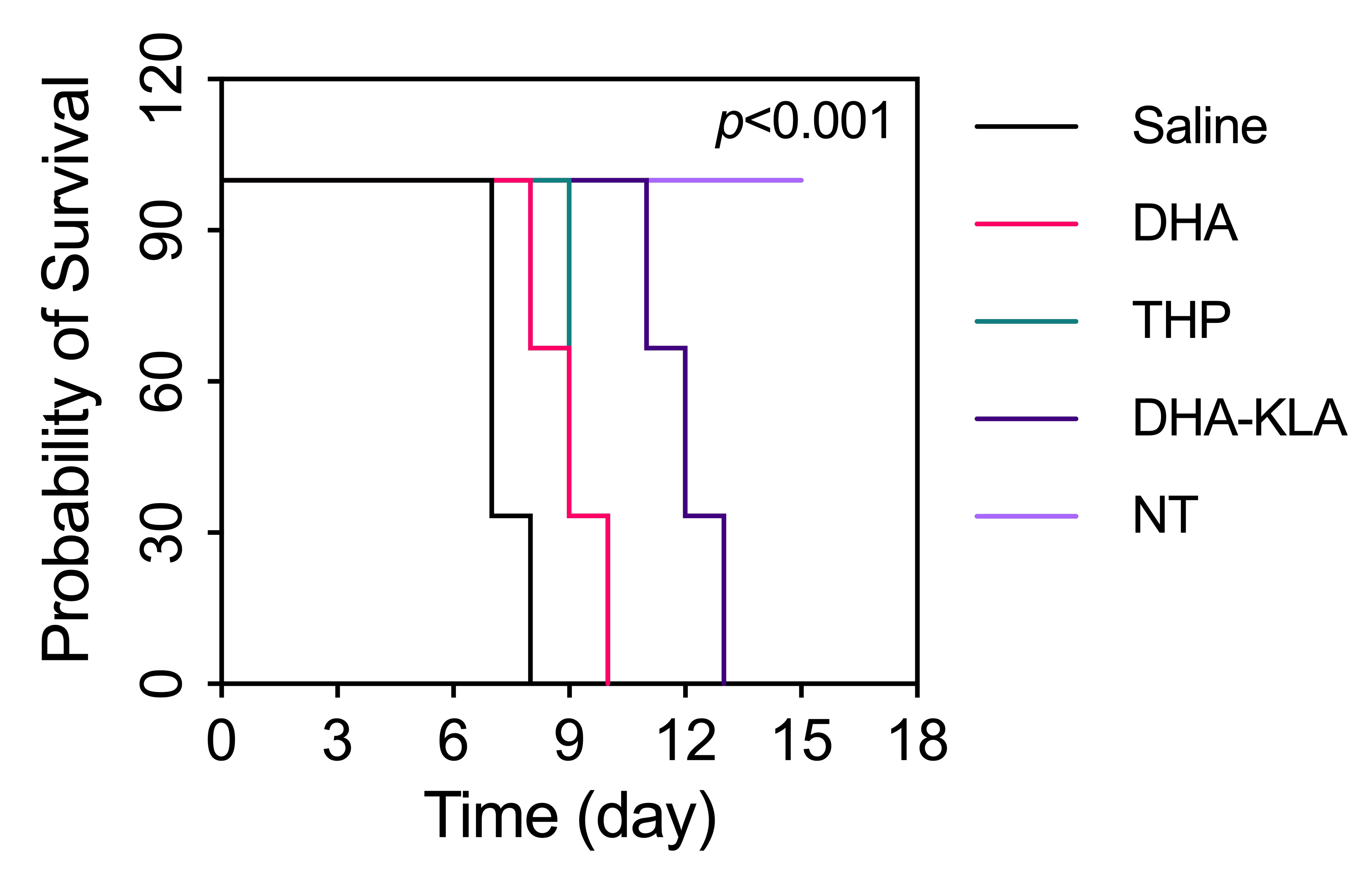


**Fig. S26** Survival curves of tumor-bearing mice with different treatments.


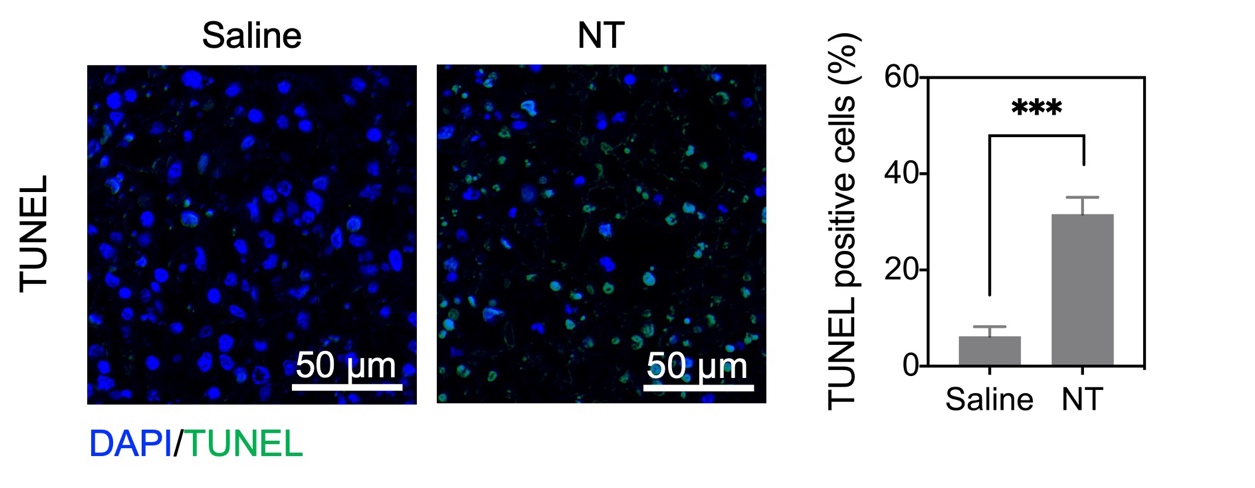


**Fig. S27** Representative immunofluorescence images and quantification of the TUNEL assay. ****p*<0.001.


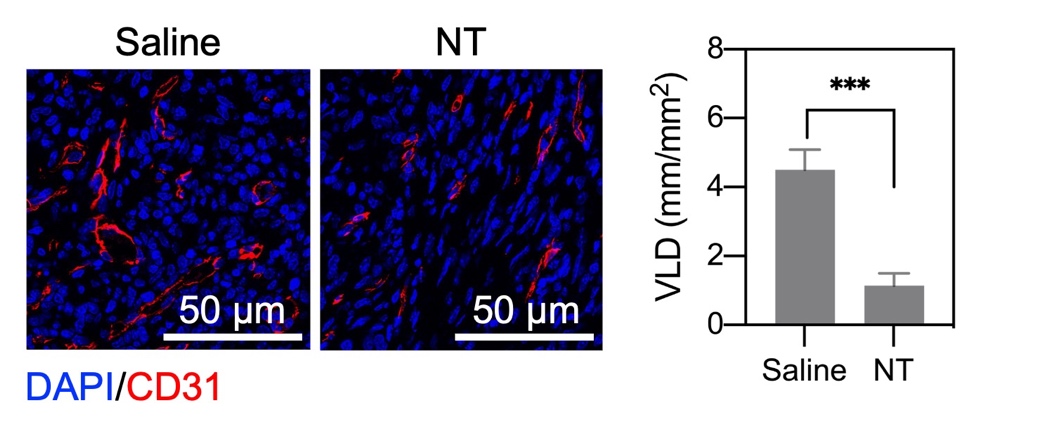


**Fig. S28** Vessel length density (VLD) was quantified as the total vessel length per unit tissue area, expressed in mm/mm^2^.


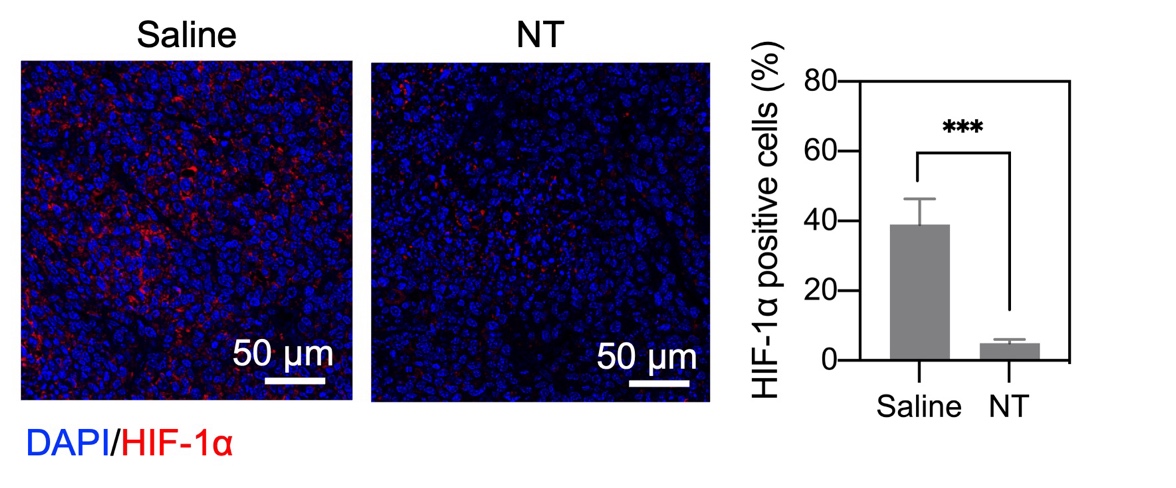


**Fig. S29** Representative immunofluorescence images and quantification of the HIF-1α. ****p*<0.001.


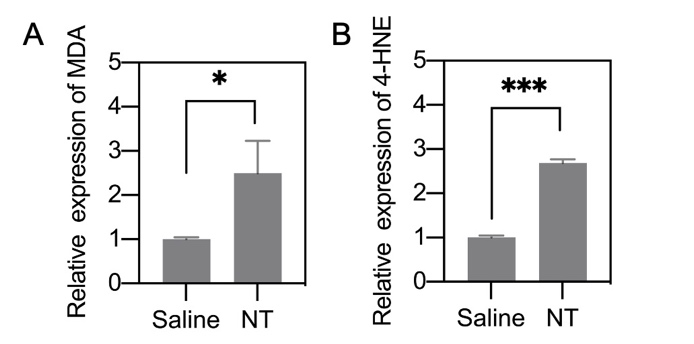


**Fig S30.** The level of (**A**) lipid peroxidation product malondialdehyde (MDA) and (**B**) 4-hydroxynonenal (4-HNE) in tumor tissues was detected using an MDA lipid oxidation assay kit and 4-HNE assay detection kit. **p*<0.05, ****p*<0.001.


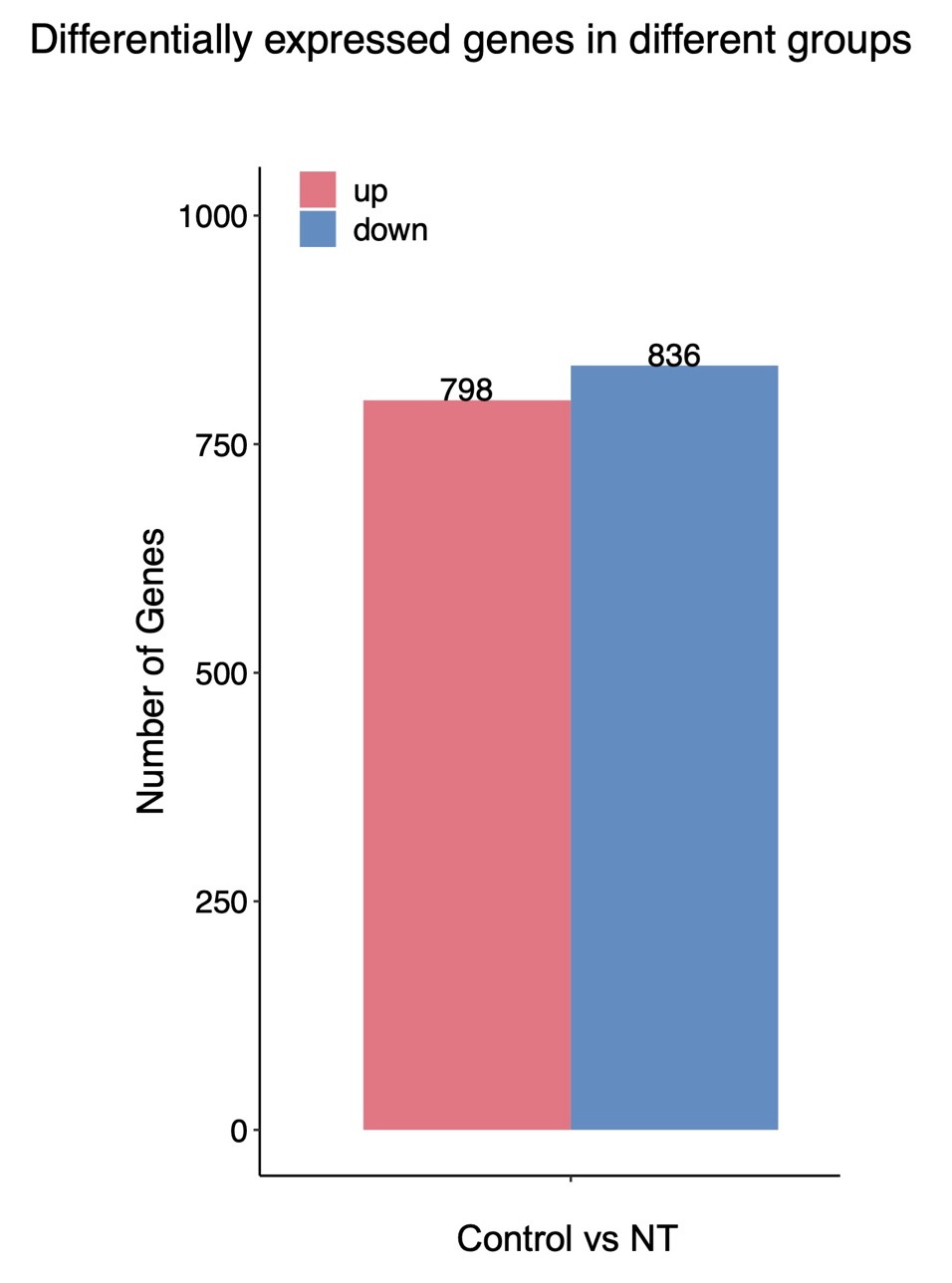


**Fig. S31** Statistics of differentially expressed genes (DEGs) between control and NT groups. Red and blue colors represent upregulated and downregulated genes, respectively.


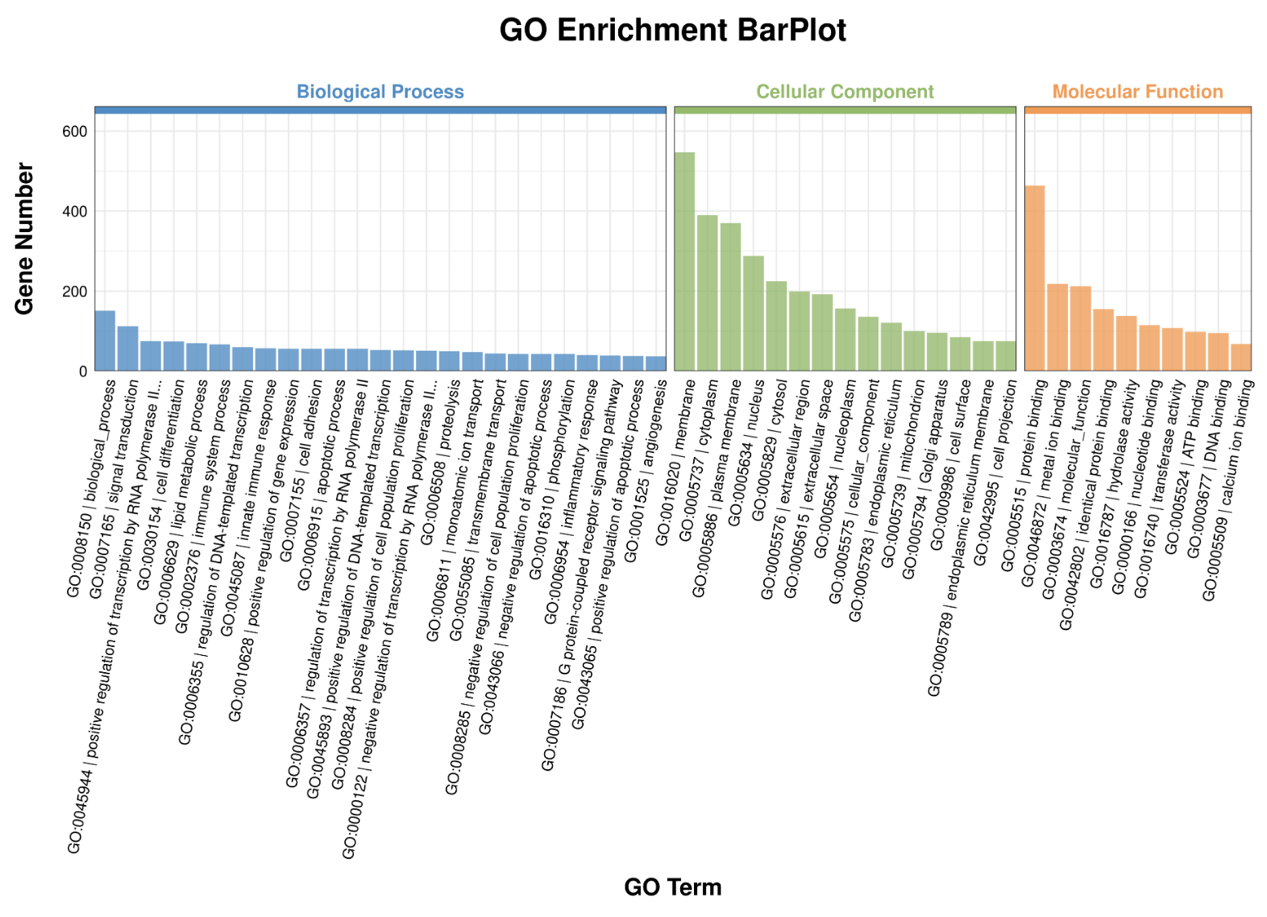


**Fig. S32** The GO enrichment bar chart shows the distribution of significantly differentially expressed genes across enriched terms in biological processes (BP), cellular components (CC), and molecular functions (MF).


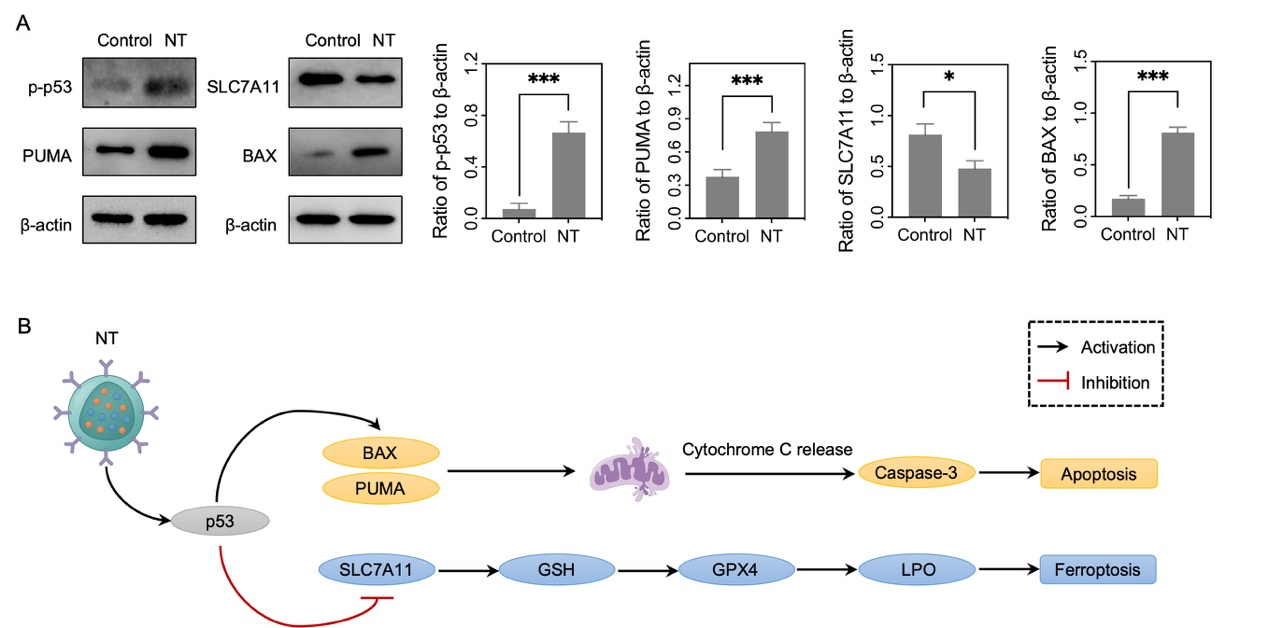


**Fig. S33 A** Western blot analysis and relative protein expression levels of phosphorylated p53 (p-p53), PUMA, Bax, and SLC7A11. **B** Schematic of the molecular pathway for NT‑induced synergistic apoptosis and ferroptosis in PDAC. **p*<0.05, ****p*<0.001.


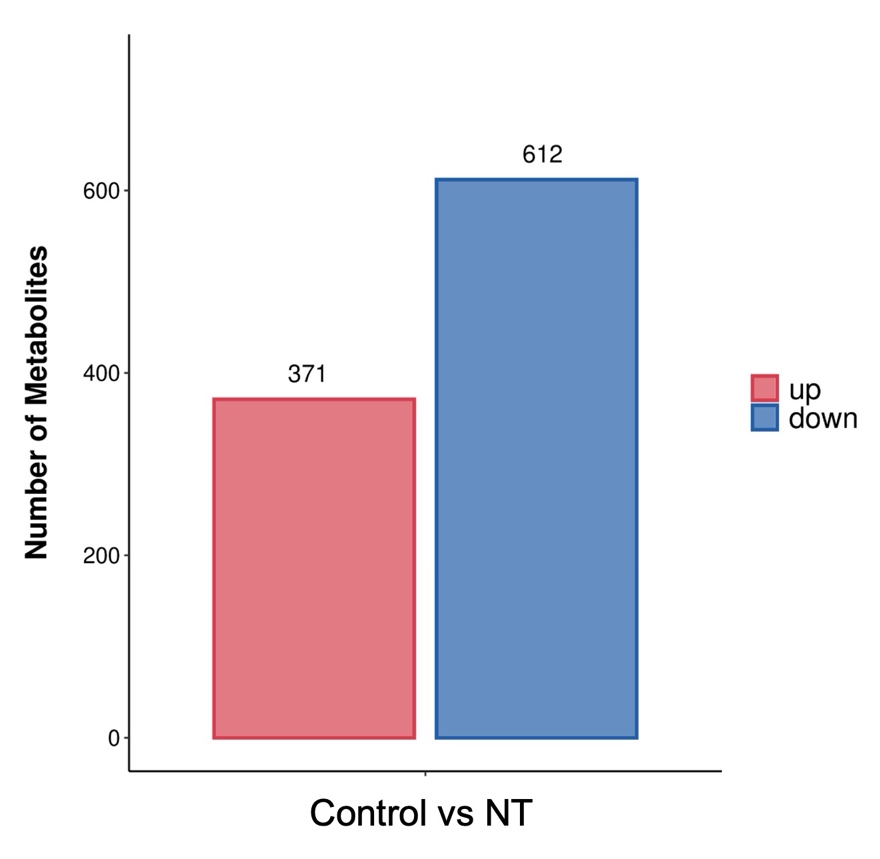


**Fig. S34** Statistics of differentially expressed metabolite ions between control and NT groups. Red and blue colors represent upregulated and downregulated ions, respectively.


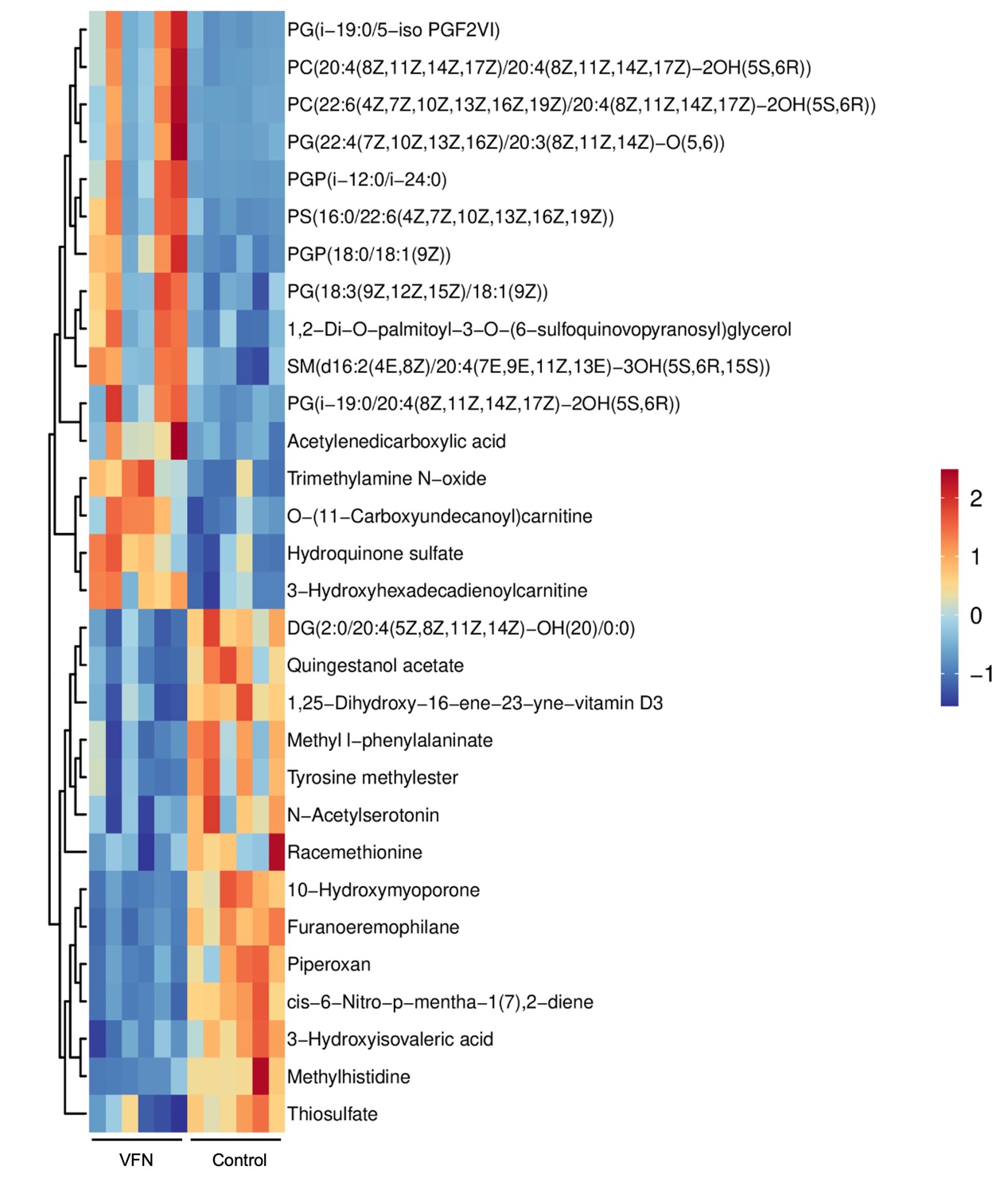


**Fig. S35** Heatmap of the top 30 differential metabolites between the NT-treated and control groups (n = 5).

**
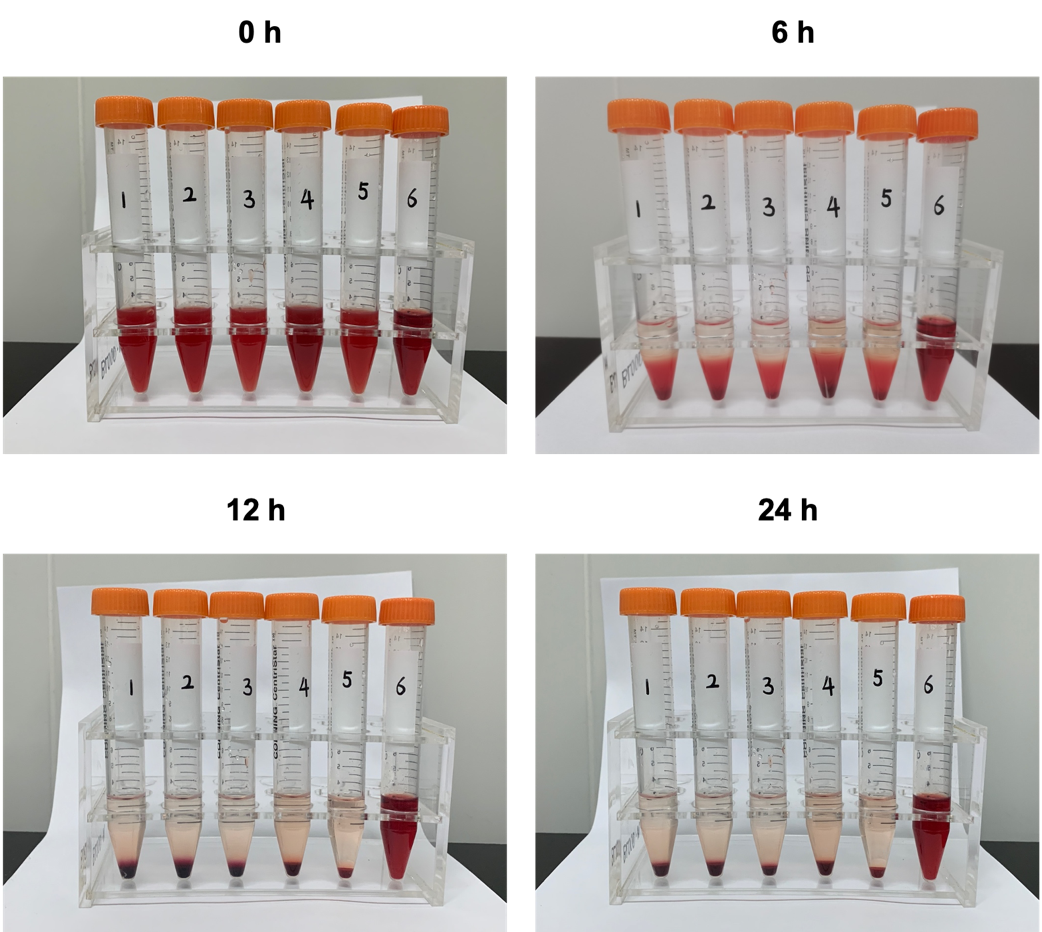
**

**Fig. S36** Representative pictures of hemolysis at the different time points were evaluated using a 2% red blood cell suspension.


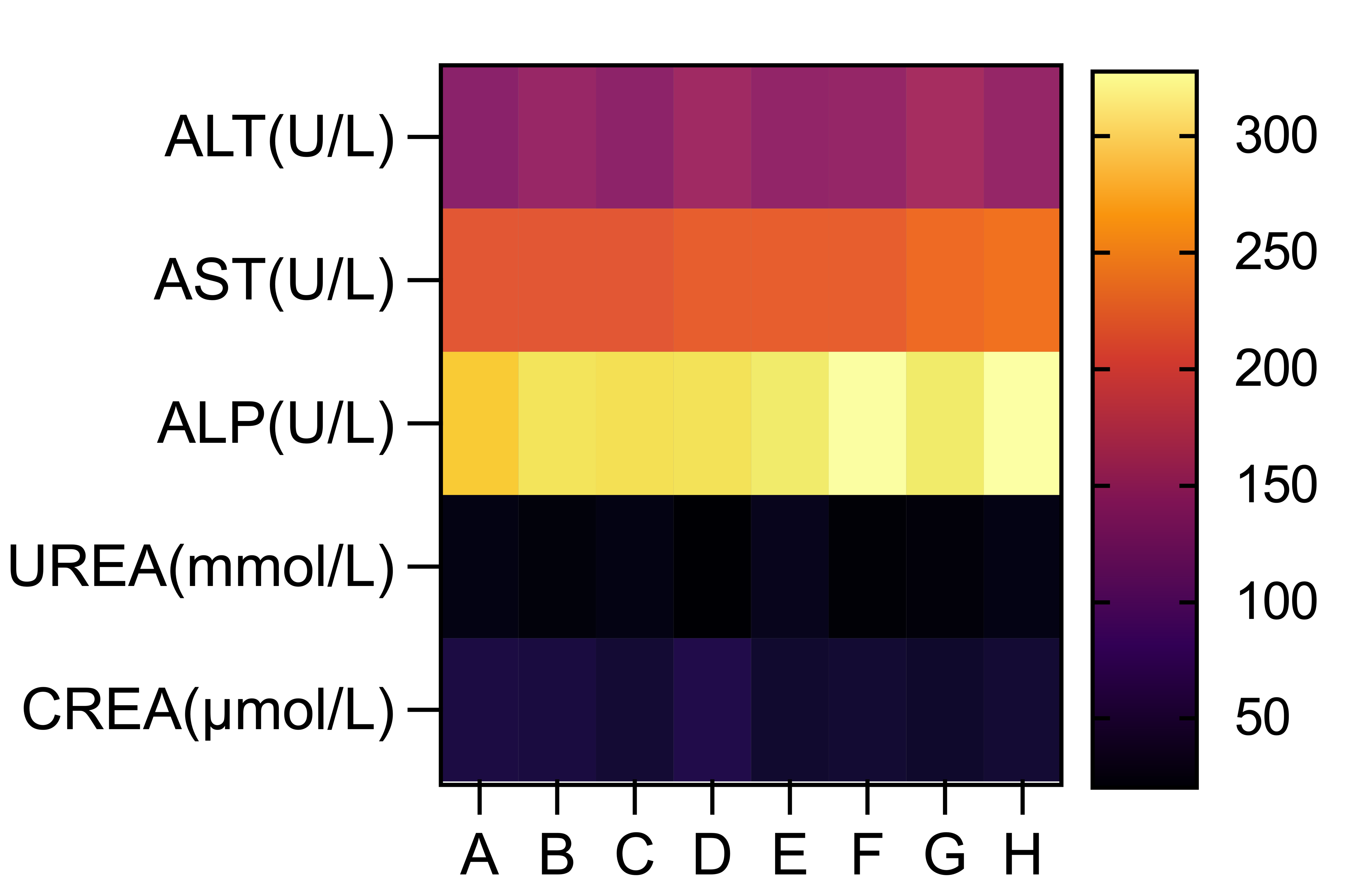


**Fig. S37** Blood biochemical analysis of nude mice at 7th or 14th days postinjection. The abscissa represents the grouping as follows. A: saline group at 7th day. B: low-dose NT group at 7th day. C: high-dose NT group at 7th day. D: high-dose DHA-KLA group at 7th day. E: saline group at 14th day. F: low-dose NT group at 14th day. G: high-dose NT group at 14th day. H: high-dose DHA-KLA group at 14th day.


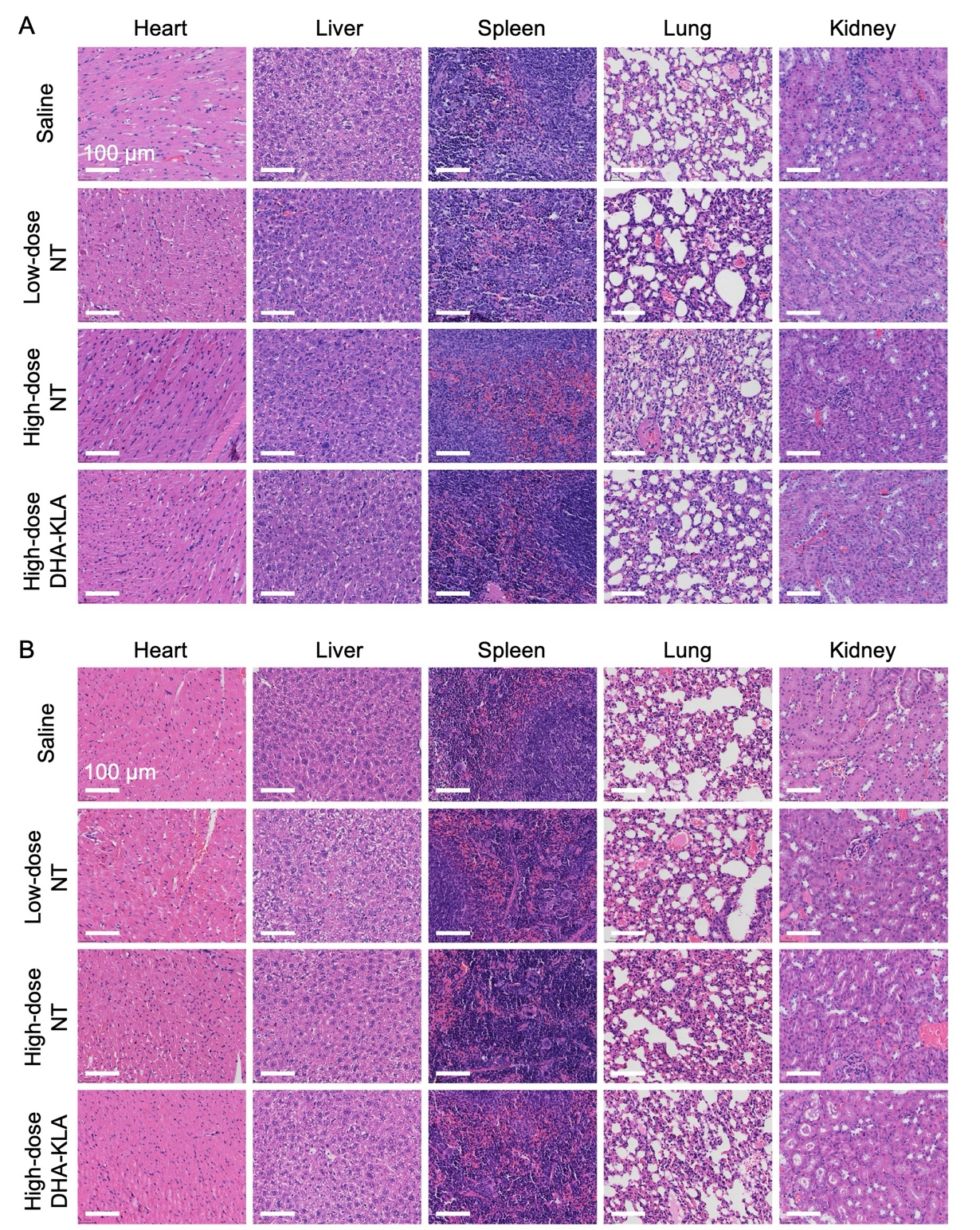


**Fig. S38** Histopathological evaluation of major organs. Representative H&E-stained sections of the heart, liver, spleen, lung, and kidney tissues collected from mice in different treatment groups at (**A**) 7 days and (**B**) 14 days post-administration.


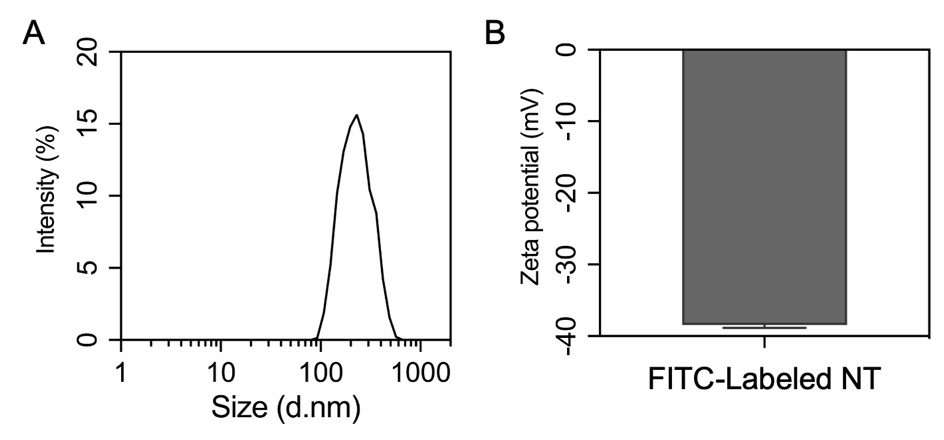


**Fig. S39. A** Hydrodynamic diameter distributions of FITC-labeled NT by DLS. **B** Zeta potential measurements of FITC-labeled NT.

**Table S1. DHA and THP peptide cumulative release from NT**

| Time (h) | Release rate of DHA (%) | Release rate of THP (%) |
| --- | --- | --- |
| 0 | 0 | 0 |
| 1 | 8.77 ± 1.49 | 5.33 ± 1.37 |
| 2 | 10.09 ± 1.99 | 8.94 ± 2.63 |
| 6 | 23.15 ± 2.11 | 27.43 ± 6.90 |
| 12 | 32.22 ± 1.14 | 46.52 ± 8.20 |
| 24 | 43.12 ± 2.22 | 58.35 ± 7.52 |
| 48 | 58.71 ± 2.72 | 71.34 ± 6.85 |
| 72 | 66.67 ± 1.06 | 78.57 ± 6.55 |

**Table S2.** **Docking scores of molecular docking of CGKRK peptide with p32 protein**

**
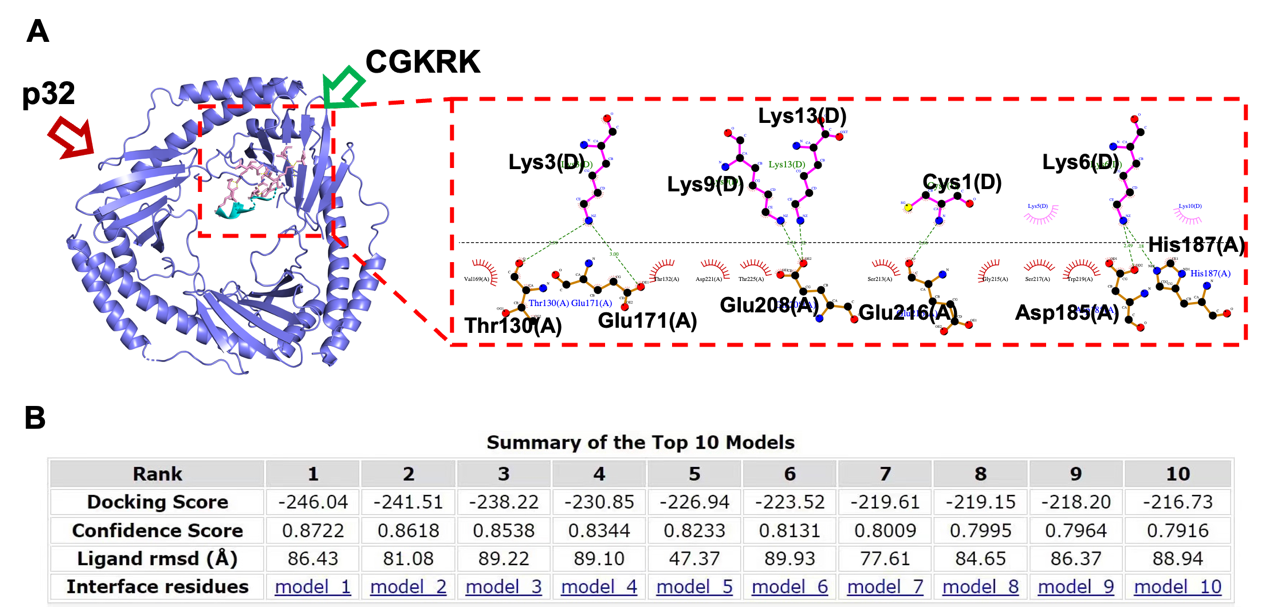
**

**Table S3.** Experimental configuration for *in vitro* hemolysis testing.

| Tube number | 1 | 2 | 3 | 4 | 5 | 6 |
| --- | --- | --- | --- | --- | --- | --- |
| 2% red blood cell suspension (mL) | 1.70 | 1.70 | 1.70 | 1.70 | 1.70 | 1.70 |
| Sterile saline (mL) | 1.65 | 1.60 | 1.65 | 1.60 | 1.70 | / |
| double-distilled water (mL) | / | / | / | / | / | 1.70 |
| NT (10 mg/mL) (mL) | 0.05 | 0.10 | / | / | / | / |
| DHA-KLA (10 mg/mL) (mL) | / | / | 0.05 | 0.1 | / | / |

**Table S4.** Hemolysis rate under different NT and DHA-KLA concentrations.

| Tube number | Final concentration (mg/mL) | Hemolysis rate (%) |
| --- | --- | --- |
| 1 | 0.147 | 1.1 |
| 2 | 0.294 | 0.7 |
| 3 | 0.147 | 0.3 |
| 4 | 0.294 | 0 |
| 5 | / | 0 |
| 6 | / | 100 |

**Table S5.** Blood routine analysis of the mice on the 7th day after DHA-KLA or NT injection.

| Blood routine parameters | Saline | Low-dose  NT | High-dose  NT | High-dose  DHA-KLA |
| --- | --- | --- | --- | --- |
| WBC (×10^9^/L) | 7.54 ± 1.69 | 10.55 ± 0.05 | 10.42 ± 1.81 | 8.71 ± 1.92 |
| RBC (×10^9^/L) | 8.35 ± 1.14 | 8.63 ± 0.57 | 7.86 ± 1.20 | 8.11 ± 1.39 |
| Hb (g/L) | 136.00 ± 19.97 | 133.50 ± 17.68 | 126.00 ± 16.97 | 129.50 ± 13.44 |
| PLT (×10^9^/L) | 1065.67 ± 260.31 | 1200.00±520.43 | 1351.00 ± 114.55 | 1416.50 ± 215.67 |
| NE% (%) | 10.20 ± 5.47 | 25.90 ± 28.71 | 7.40 ± 1.27 | 14.90 ± 5.23 |
| LYM% (%) | 65.37 ± 8.67 | 48.80 ± 25.31 | 67.60 ± 9.90 | 53.75 ± 12.66 |
| MON% (%) | 11.17 ± 1.03 | 9.10 ± 7.35 | 12.45 ± 7.71 | 12.90 ± 4.10 |
| BAS% (%) | 13.27 ± 4.52 | 16.15 ± 3.89 | 12.55 ± 0.92 | 18.45 ± 3.32 |
| NE# (%) | 0.72 ± 0.25 | 2.74 ± 3.04 | 0.78 ± 0.27 | 1.25 ± 0.18 |
| LYM# (%) | 4.96 ± 1.36 | 5.14 ± 2.64 | 6.96 ± 0.19 | 4.80 ± 2.14 |
| MON# (%) | 0.83 ± 0.15 | 0.96 ± 0.77 | 1.37 ± 1.03 | 1.09 ± 0.11 |
| BAS# (%) | 1.03 ± 0.53 | 1.71 ± 0.42 | 1.32 ± 0.33 | 1.58 ± 0.06 |
| HCT (%) | 0.49 ± 0.06 | 0.47 ± 0.07 | 0.45 ± 0.06 | 0.46 ± 0.05 |
| MCV (fL) | 59.00 ± 2.26 | 54.10 ± 4.81 | 57.35 ± 1.06 | 57.50 ± 3.96 |
| MCHC (g/L) | 16.30 ± 0.89 | 15.45 ± 1.06 | 16.05 ± 0.35 | 16.05 ± 1.06 |
| MCH (pg) | 276.00 ± 5.29 | 285.50 ± 6.36 | 280.00 ± 0.00 | 279.50 ± 0.71 |
| RDWCV (%) | 18.30 ± 0.35 | 20.70 ± 3.39 | 18.30 ± 3.54 | 18.20 ± 1.84 |
| MPV (fL) | 7.70 ± 0.44 | 7.55 ± 0.49 | 7.85 ± 0.21 | 7.85 ± 0.35 |

**Table S6.** Blood routine analysis of the mice on the 14th day after DHA-KLA or NT injection.

| Blood routine parameters | Saline | Low-dose  NT | High-dose  NT | High-dose  DHA-KLA |
| --- | --- | --- | --- | --- |
| WBC (×10^9^/L) | 9.45 ± 1.94 | 6.71 ± 1.98 | 6.87 ± 1.77 | 6.33 ± 3.15 |
| RBC (×10^9^/L) | 8.37 ± 0.29 | 9.89 ± 0.81 | 9.93 ± 0.72 | 9.61 ± 0.11 |
| Hb (g/L) | 134.67 ± 0.58 | 154.00 ± 7.07 | 165.00 ± 8.49 | 150.50 ± 2.12 |
| PLT (×10^9^/L) | 1085.33 ± 247.87 | 1089.00 ± 82.02 | 701.50 ± 729.03 | 1359.50 ± 457.50 |
| NE% (%) | 9.83 ± 2.83 | 8.40 ± 3.39 | 7.20 ± 0.85 | 8.10 ± 1.70 |
| LYM% (%) | 70.77 ± 3.54 | 67.90 ± 1.13 | 66.40 ± 6.65 | 73.00 ± 1.56 |
| MON% (%) | 6.13 ± 2.19 | 9.60 ± 0.85 | 10.20 ± 12.02 | 4.15 ± 0.64 |
| BAS% (%) | 13.20 ± 1.75 | 13.80 ± 0.99 | 15.90 ± 4.81 | 14.75 ± 0.49 |
| NE# (%) | 0.93 ± 0.31 | 0.60 ± 0.40 | 0.50 ± 0.18 | 0.54 ± 0.36 |
| LYM# (%) | 6.73 ± 1.72 | 4.55 ± 1.27 | 4.62 ± 1.63 | 4.60 ± 2.20 |
| MON# (%) | 0.56 ± 0.13 | 0.64 ± 0.13 | 0.60 ± 0.64 | 0.26 ± 0.09 |
| BAS# (%) | 1.23 ± 0.09 | 0.92 ± 0.21 | 1.14 ± 0.62 | 0.94 ± 0.49 |
| HCT (%) | 0.46 ± 0.01 | 0.54 ± 0.05 | 0.57 ± 0.02 | 0.51 ± 0.01 |
| MCV (fL) | 55.33 ± 3.37 | 54.15 ± 1.06 | 57.35 ± 1.91 | 52.80 ± 1.27 |
| MCHC (g/L) | 16.13 ± 0.55 | 15.60 ± 0.57 | 16.65 ± 0.35 | 15.65 ± 0.07 |
| MCH (pg) | 291.67 ± 9.07 | 288.00 ± 15.56 | 290.00 ± 2.83 | 296.50 ± 7.78 |
| RDWCV (%) | 18.77 ± 0.51 | 21.30 ± 1.84 | 19.85 ± 1.06 | 20.10 ± 0.99 |
| MPV (fL) | 7.33 ± 0.06 | 7.05 ± 0.21 | 7.25 ± 0.07 | 7.20 ± 0.42 |
